# Targeting the MDK/c‐Myc complex to overcome temozolomide resistance in glioma

**DOI:** 10.1002/ctm2.70359

**Published:** 2025-06-04

**Authors:** Xiaonan Xi, Xiaojing Ding, Qianqian Wang, Ning Liu, Bangmao Wang, Genbei Wang, Weilong Zhong, Yaxin Lu

**Affiliations:** ^1^ State Key Laboratory of Medicinal Chemical Biology Nankai University Tianjin China; ^2^ College of Pharmacy Nankai University Tianjin China; ^3^ Pharmacology & Toxicology Research Center National Key Laboratory of Chinese Medicine Modernization Tasly Pharma Co., Ltd. Tianjin China; ^4^ Department of Gastroenterology and Hepatology Tianjin Medical University General Hospital Tianjin Institute of Digestive Diseases Tianjin Key Laboratory of Digestive Diseases Tianjin China; ^5^ College of Chemistry Nankai University Tianjin China

**Keywords:** ACT001, drug resistance, glioma, MDK, protein complex

## Abstract

**Background:**

Temozolomide (TMZ), which is an alkylating agent, is the standard chemotherapeutic drug used for glioma treatment. However, the development of resistance to TMZ limits its efficacy. Thus, identifying novel therapeutic targets is necessary.

**Methods:**

In this study, the levels of midkine (MDK) and c‐Myc expression in glioma patient samples downloaded from TCGA were analyzed. Their interactions were also demonstrated through microthermometry and immunocoprecipitation. Furthermore, proteomics technology and Western blot showed that MDK interacted with c‐Myc and influenced its ubiquitination, thereby activating a prosurvival signalling pathway and epithelial–mesenchymal transition mechanism, which contributed to TMZ resistance. To target the MDK/c‐Myc complex, we screened for a small‐molecule inhibitor (ACT001) that specifically disrupts the interaction between MDK and c‐Myc. Treatment with ACT001 greatly sensitized TMZ‐resistant glioma cells to TMZ, promoting cell death and inhibiting cell proliferation. Moreover, combination therapy with ACT001 and TMZ showed synergistic effects that inhibit tumour growth in glioma xenograft models and glioma in situ models.

**Results:**

ACT001 facilitated the degradation of c‐Myc by focusing on the MDK/c‐Myc complex and controlled the Wnt/β‐catenin signalling pathway via MDK, ultimately halting the advancement of glioma. When combined with TMZ, ACT001 showed good therapeutic potential for the treatment of glioma.

**Conclusion:**

Focusing on the MDK/c‐Myc complex could be an effective approach to combat resistance to TMZ in glioma. Therapy with ACT001 may be a novel approach to improve the efficacy of TMZ‐based chemotherapy in patients with glioma. Further preclinical and clinical studies are warranted to validate the therapeutic potential of targeting the MDK/c‐Myc complex in glioma treatment.

## Graphical abstract

ACT001 disrupts the MDK/c‐Myc complex, enhancing c‐Myc ubiquitination and overcoming temozolomide resistance in glioma. Combining ACT001 with temozolomide synergistically inhibits tumour growth and progression, offering a promising therapeutic strategy for glioma patients, particularly those with high MDK expression.

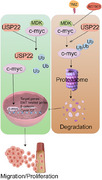

## 1 INTRODUCTION

Glioma, the most prevalent primary brain tumour, makes up 27% of all central nervous system tumours and 80% of malignant tumours, particularly glioblastoma, which is known for its high invasiveness and rapid recurrence.^1^, ^2^ Despite developments in maximal surgical resection, radiation therapy, and chemotherapy, the overall survival (OS) rate of patients with glioma is around 15 months.^3^, ^4^, ^5^

Midkine (MDK) is a secretory heparin‐binding growth factor encoded by a gene located at the 11p11.2 locus on chromosome 11. A mature MDK molecule has a relatively small molecular weight of only 14 kDa.^6^ MDK is highly expressed in various types of cancer, and it plays a critical role in the tumour microenvironment by promoting tumour cell proliferation, invasion, metastasis, and angiogenesis, as well as exerting anti‐apoptotic effects.^7^, ^8^, ^9^, ^10^ These effects are mediated through the activation of downstream signalling pathways, such as Notch1/p‐JNK, via binding to specific receptors or receptor complexes.^11^, ^12^, ^13^, ^14^, ^15^, ^16^, ^17^ In glioblastomas, MDK is highly expressed and associated with poor prognosis.^9^, ^15^ Previous studies have demonstrated that MDK contributes to tumour progression and therapy resistance, including resistance to temozolomide (TMZ), through mechanisms involving cancer stem cell maintenance and epithelial–mesenchymal transition (EMT).^15^ Despite these findings, the precise molecular mechanism by which MDK promotes glioblastoma progression and resistance remains unclear. Furthermore, although MDK has been extensively studied in glioblastomas, its interaction with key oncogenic factors and its role in regulating critical signalling pathways, such as Wnt/β‐catenin, require further investigation. Addressing these gaps could provide new insights into the development of targeted therapies for glioma.

The *c‐Myc* gene belongs to the *Myc* oncogene family, and it can be found on chromosome 8q24 in humans.^18^, ^19^ The c‐Myc protein regulates nuclear transcription factor activity, plays a role in DNA proliferation, and promotes the abnormal differentiation, invasion, and migration of tumour cells.^20^ The c‐Myc protein serves as a transcription factor by forming a heterodimer with MAX protein, binding to DNA core sequences, and controlling the activity of target genes. c‐Myc can control the levels of proteins involved in the cell cycle, such as cyclin, lactate dehydrogenase A, transcription factor E2F, and the apoptosis‐related protein Bax, which in turn influences cellular behaviour.^21^ Under normal physiological conditions, the expression of the *c‐Myc* gene is strictly regulated. In human tumour tissues, the gene is excessively amplified, abnormally expressed, and rearranged.^22^ These processes are involved in the regulation of tumour cell proliferation, metabolism, differentiation, and cell cycle.^23^

Although drug therapy is considered a useful tool in managing the progression of glioma, the options for treating glioma are currently restricted, and they come with unwanted side effects. In addition, many drugs struggle to penetrate the blood–brain barrier.^24^, ^25^, ^26^ Therefore, the development of effective targeted drugs for glioma is urgently necessary. ACT001, which is a parthenolide derivative with high anticancer activity,^27^, ^28^ has been shown to cross the blood–brain barrier, achieving a brain tissue concentration 1.8 times higher than that in blood, which highlights its potential for treating brain tumours. Preclinical and clinical studies have also demonstrated the tolerability and safety of ACT001 in glioma treatment.^29^, ^30^ Despite multiple studies reporting the target sites of ACT001, this parthenolide derivative is a multi‐target compound, and its anti‐tumour effects are the result of multifaceted synergy.

MDK and c‐Myc have been implicated as important regulatory factors in the growth and progression of glioma. The disruption of the interaction between MDK and c‐Myc has potential therapeutic effects on glioma. Studying the impact of ACT001 on the sensitivity of TMZ can facilitate the development of effective combination therapy regimens that can improve treatment outcomes and survival rates for patients with glioma.

## 2 MATERIALS AND METHODS

### 2.1 Source of clinical samples

Clinical data were collected from 60 cases of glioma specimens from patients who underwent glioma resection surgery at Tianjin Medical University General Hospital. The samples included 39 male and 21 female patients, aged between 20 and 78 years, all classified as WHO grade IV. The inclusion criteria were as follows: (1) postoperative pathology‐confirmed glioma; (2) patients had surgical indications and voluntarily underwent surgery; (3) no prior radiotherapy, chemotherapy, or immunotherapy. The exclusion criteria included patients with other malignant tumours, those who had undergone secondary glioma surgery, and patients with missing medical records or those who died during the perioperative period. Furthermore, 10 cases of associated para‐cancerous brain tissue samples located over 1 cm from the tumour were collected from patients undergoing neurosurgery. This study was approved by the Medical Ethics Committee, and informed consent was obtained from the patients and their families.

### 2.2 Analysis of WGCNA expression, survival time, and prognosis

Data from 156 patients with different types of glioma were collected and downloaded from the TCGA database. The data included a variety of clinical information (gender, age, and postoperative treatment modalities, including radiotherapy and chemotherapy) as well as pathological information (histopathology and WHO grade) and molecular pathological information. Follow‐up information (OS time) and MDK mRNA sequencing data for the corresponding patients were also included. To compare MDK mRNA expression in patients with glioma, the Oncomine database online was used (https://www.oncomine.org/) to analyze the differences between cancer and normal tissues. Next, a Kaplan–Meier curve was generated using the Kaplan–Meier plotter web tool (https://Kaplan‐Meier plotter) to examine the correlation between MDK mRNA levels in brain tissue and OS of individuals with brain cancer.

### 2.3 Cell culture

U251, U87, SF126, U118MG, BT‐325, and SHG44 were supplied by Accendatech Co., Ltd. HEB was acquired from BeNa Culture Collection in China. TMZ‐resistant U118MG cells were prepared in our laboratory in accordance with the methods described in the literature.^31^ The concentrations of TMZ used for screening were 2.5, 5, 10, 20, 40, 80, 160, and 320 µM. Each concentration was maintained until the cells grew steadily for six generations. Following a minimum of 10 months of induction, the resistant cell lines were successfully selected and named U118MG‐R. HEB, U251, U87, SF126, and U118MG cells were grown in Dulbecco's modified Eagle medium (C11995500BT, Gibco). BT‐325 and SHG44 were cultured in RPMI1640 (C11875500BT, Gibco). Each medium was enhanced with 10% fetal bovine serum (04‐001‐1ACS, Biological industries), 100 IU/mL penicillin, and .1 mg/mL streptomycin (15140‐122, Gibco) and placed in a 5% CO_2_‐humidified incubator at 37°C. All the cell lines were correctly identified by STR, and no mycoplasma contamination was observed.

### 2.4 Plasmid and viral transfection and construction of stable cell lines

A recombinant sh‐MDK lentivirus was purchased from the OBiO Technology Corp., Ltd. MDK overexpression plasmid was purchased from Sino Biological, Inc. (HG10247‐ACR). U251, U87, SF126, U118MG, BT‐325, and SHG44 cells were placed in six‐well dishes for cell culture. The recombinant sh‐MDK lentivirus was added to the U251, SF126, and U118MG cells and incubated overnight. Next, the medium with the lentivirus was discarded, and a fresh medium was added. After 24 h, 3 µg/mL puromycin (P8230, Solarbio) was added to screen stable MDK knockdown cell lines (shMDK). The MDK overexpression plasmid was transfected using Lipofectamine 2000 (Invitrogen) in accordance with the manufacturer's instructions. Hygromycin (IH0160, Solarbio) was used to screen stable overexpressed MDK cell lines (OEMDK). shMDK SF126‐Luc, shMDK U118MG‐Luc, and OE‐MDK U87‐Luc cells expressing luciferase were obtained by transfecting a luciferase overexpression lentivirus labelled as blasticidin (purchased from OBiO Technology Corp., Ltd.) into shMDK SF126, shMDK U118MG, and OE‐MDK U87 cells. The screening process for other stable cell lines follows the same protocol, with resistance being screened in accordance with the guidelines provided by the manufacturer.

### 2.5 Quantitative real‐time PCR

The MDK qPCR primer pair was purchased from Sino Biological, Inc. (HP100297). Precooled phosphate‐buffered saline (PBS) was used to rinse the cells. Total RNA was extracted in accordance with the guidelines provided by the manufacturer (DP430, Tiangen). After determining the purity and concentration of RNA, an RT‐PCR kit (KR123, Tiangen) was used for PCR quantification. GAPDH was used as an internal reference gene. The primer sequence of GAPDH was as follows: (F) 5′‐GTCTCCTCTGACTTCAACAGCG‐3′, (R) 5′‐ACCACCCTGTTGCTGTAGCCAA‐3′. The relative expression level of the target gene was calculated by using the 2^−△△Ct^ method.

### 2.6 Western blotting assay

Total protein was extracted using RIPA buffer (R0020, Solarbio) with protease inhibitor cocktails (P1005, Beyotime). The protein concentration was measured using a BCA protein assay kit (PC0020, Solarbio). An appropriate amount of protein was heated to 100°C for 5 min. Following denaturation, sodium dodecyl sulfate‐polyacrylamide gel electrophoresis (SDS‐PAGE) was performed. After electrophoresis, the proteins were transferred onto a PVDF membrane and incubated in 5% skim milk for 1 h. Primary antibodies targeting MDK (1:1000, 11009‐1‐AP, Proteintech), GAPDH (1:8000, 10494‐1‐AP, Proteintech), c‐Myc (1:1000, 10828‐1‐AP, Proteintech), ubiquitinylated proteins (1:1000, 04–263, Millipore), cyclin D1 (1:1000, AF0931, Affinity), cyclin D1 (1:1000, 60186‐1, Proteintech), β‐catenin (1:1000, AF6266, Affinity), E‐cadherin (1:1000, AF0131, Affinity), N‐cadherin (1:1000, AF5239, Affinity), vimentin (1:1000, AF7013, Affinity), MMP‐2 (1:1000, AF5330, Affinity), and MMP‐9 (1:1000, AF5228, Affinity) were added and left to incubate overnight at 4°C. The membranes were washed three times with TBST for 5 min. Next, a secondary goat antirabbit (SA00001‐2, Proteintech) or goat antimouse HRP‐IgG (SA00001‐1, Proteintech) was added and left to incubate at 37°C for 1 h. Afterwards, the membranes were rinsed with TBST, and an ECL luminescence reagent (SQ202, Epizyme) was added to the membranes for visualization.

### 2.7 EdU assay

Confocal dishes were inoculated with cells in the logarithmic growth phase. After the cells were cultured in groups in accordance with the experimental plan, the BeyoClick EdU‐555 cell proliferation detection kit (C0075, Beyotime) was used for cell labelling. Images were obtained using an LSM800 laser confocal microscope (Zeiss).

### 2.8 Cell invasion assay

The invasion assay was performed as follows: Matrigel (354234, Corning) was placed in an ice bath at 4°C until the ice melted. The Matrigel was mixed with the appropriate incomplete medium in a 1:1 ratio and then placed on ice. Transwell chambers were placed in 24‐well plate holes, and 50 µL of Matrigel was added to each chamber. Then, the Transwell chambers were placed in the incubator for more than 30 min to allow the Matrigel to fully solidify. Afterwards, 200 µL of cell suspension (prepared with an incomplete medium) was added to the upper chamber of each chamber, and a complete medium (700 µL/well) was added to the lower chamber and placed in the incubator. The Matrigel was gently wiped using a cotton swab after 48 h, and the medium in the chamber was removed. The chamber was washed three times with PBS, followed by fixation with precooled methanol for 15 min. Next, the chamber was rinsed three times with PBS, followed by staining with crystal violet for around 30 min. Afterwards, it was washed with PBS until the crystal violet floating colour was no longer visible. The bottom membrane of the chamber was collected, sealed with neutral gum, and observed using an inverted microscope, with five fields of view being randomly selected and counted.

### 2.9 Colony formation assay

The cells were inoculated in a 12‐well plate with 300 cells per well. The cells were placed in an incubator and cultured for 10 days. Following the removal of the cell media, the cells were washed with PBS and then treated with 4% paraformaldehyde for 30 min. Staining was conducted for 30 min using a crystal violet solution. The residual staining solution was fully washed away, and the number of clones formed was counted under an inverted microscope.

### 2.10 CCK‐8 assay

The CCK‐8 cell proliferation and cytotoxicity assay kits were acquired from Solarbio (CA1210). A total of 4000 cells were added to each well of 96‐well plates for inoculation. The cells in the experimental group were exposed to treatment for either 48 or 72 h. Following the addition of 10 µL of CCK‐8 solution, the cells were placed in an incubator for an additional 4 h. The survival rate of the cells was determined by measuring the absorbance at 450 nm using a microplate reader (Thermo Scientific Varioskan Flash). The trial was conducted at least three times.

### 2.11 Tumour xenograft model

Five‐week‐old female nude Balb/C mice were acquired from Charles River in Beijing, China, and housed at the animal resource centre in Nankai. The ethics committee of Nankai University approved all experiments involving animals. Tumour cells (2 × 10^6^) were inoculated in the right axilla of each nude mouse with a total volume of .1 mL (including .05 mL of Matrigel). Tumour growth was observed after inoculation. Finally, a tumour volume of approximately 100 mm^3^ was used as the standard for screening, and animals with extremely large or small tumour volumes were excluded. The body weight and tumour volume were measured every 3 days. The tumour tissues were excised, and their weights were measured at the end of the experiment. Formalin was used to fix the tumour tissues, which were then embedded in paraffin and cut into 5 µm sections for immunohistochemical analysis. In the drug therapy trial, ACT001 (dissolved with normal saline and intragastric) was administered at a dosage of 200 mg/kg six times a week for 5 weeks. TMZ (25 mg/kg; T1178, TargetMol) was given weekly for five doses in total. The control group was given an equal quantity of drug vehicles.

### 2.12 Mouse brain orthotopic transplantation tumour model

Six‐week‐old male nude Balb/C mice were acquired from Charles River in Beijing, China, and housed in the animal resource centre. The ethics committee of Nankai University approved all experiments involving animals. U118MG‐Luc‐expressing luciferase was obtained by transfecting the luciferase overexpression lentivirus blasticidin into U118MG cells. Under specific pathogen‐free conditions, the mice were anaesthetized using 1.25% tribromoethanol (Aibei Biotechnology Co., Ltd.), and then their heads were secured on a brain stereotactic device in a prone position. Following the disinfection of the scalp with 75% ethanol, a vertical cut was performed to expose the fontanel located after the eye slit along the centre of the skull. A 1 mm‐diameter skull drill was used to create a hole located 2 mm anterior to the midpoint of the fontanel and 2 mm to the right of the sagittal suture. Then, the U118MG‐Luc cells were injected with 5 µL of cell suspension (1.3 × 10^5^ cells) using a microinjector. The depth of injection was 3.5 mm from the skull surface. The needle was pulled back slightly by approximately .5 mm before injection, and then the tumour cell was injected slowly at a rate of .5 µL/min. After 2 min, the needle was removed gradually, and the bone hole was sealed using bone wax. The surgical field was rinsed with normal saline, and the scalp was sutured. Finally, the wound was disinfected with iodine. After the operation, the nude mice were placed on a 37°C thermostatic plate for heat preservation and then returned to the cage after recovery. The dose and method of administration were the same as those in the “Tumour xenograft model” and “Immunohistochemistry” sections. Before imaging, each mouse was intraperitoneally injected with 100 µL of 15 mg/mL D‐luciferin potassium (MB1834, Meilunbio). The imaging instrument was IVIS Spectrum (PerkinElmer USA). The imaging method is suitable for subcutaneous tumours.

### 2.13 Pulmonary metastasis

SF126 and U118MG shMDK cells, along with U87 OEMDK cells, were injected via the tail vein. Each mouse was injected with 100 µL of 2 × 10^6^cells. The tumour size and body weight were measured every 3 days. At the end of the animal experiment, the lung tissues of the nude mice were collected, photographed, fixed using paraformaldehyde, embedded in paraffin, and cut into slices. In accordance with the manufacturer's procedures, the hematoxylin–eosin (HE) stain kit (G1120, Solarbio) was applied to stain lung tissue.

### 2.14 Co‐immunocoprecipitation

In accordance with the guidelines provided by the manufacturer, MDK (HG10247‐NF, Sino Biological) and c‐Myc overexpression plasmids (HG11346‐ACR, Sino Biological) were introduced into cells using lipo2000 (11668019, Invitrogen). Cell lysates were obtained 48 h post‐transfection by incubating the cells in IP lysis buffer (87787, Pierce) with protease inhibitor cocktails (Beyotime) for 20 min at 4°C. Next, the cell extracts were centrifuged at 14000*g* at 4°C for 20 min. The control or primary antibody along with protein A/G magnetic beads (HY‐K0202, MCE) was mixed in a 3D rotating mixer at room temperature for 30 min, followed by magnetic separation on a magnetic rack to collect the beads. After washing four times with binding/washing buffer (1× PBS + 0.5% Tween‐20), the magnetic bead/antibody complex was combined with 500 µg of protein supernatant (1 mg/mL) and then incubated at 4°C on a 3D rotating mixer for 2 h. Then, the mixture was subjected to magnetic separation, resulting in the removal of the supernatant. After the magnetic bead/antibody/antigen complex was fully washed with binding or washing buffer four times, 50 µL of 1× loading buffer was added to the magnetic bead. The mixture was mixed evenly and boiled for 5 min. The supernatant was then collected for SDS‐PAGE and Western blot.

### 2.15 Silver staining was performed, and interacting proteins were identified through mass spectrometry

FLAG‐MDK (HG10247‐NF, Sino Biological) was stably expressed in SF126 and U118MG cells. Then, lysates were prepared by incubating the cells with RIPA buffer (Solarbio) supplemented with a protease inhibitor cocktail (Beyotime). The flag‐tag protein IP assay kit with magnetic beads (P2181S, Beyotime) was utilized to purify the MDK protein labelled with the flag tag, in accordance with the guidelines provided by the manufacturer. Following SDS‐PAGE, the obtained sample was dyed using a fast silver stain kit (P0017S, Beyotime). The specific bands on the SDS PAGE were cut. After the strip was washed and decolourized, DTT and IAA were added to promote the alkylation of disulfide bonds. Finally, trypsin (V5111, Promega) was added. Peptides were isolated, concentrated using vacuum centrifugation, dissolved in 1‰ formic acid solution, and analyzed using an Orbitrap Fusion Tribrid mass spectrometer from Thermo. The original spectral data were analyzed using Proteome Discoverer 2.2 (Thermo).

### 2.16 Preparation, detection, and data analysis of proteomic samples

MDK siRNA (SC‐39711) and the negative control siRNA (sc‐37007) were obtained from Santa Cruz Biotechnology, Inc. Transfection was conducted using lipo2000 (11668019, Invitrogen) in accordance with the instructions provided by the manufacturer. After transfection for 48 h, U118MG cells were collected. Proteomic samples were prepared, and data analysis for mass spectrometry was performed as previously described.^28^

### 2.17 Microscale thermophoresis

The interaction between c‐Myc and MDK was measured by Monolith NT.115 (NanoTemper). Recombinant human MDK protein was purchased from Zeye Biotechnology Corporation (ZY631H02P, Shanghai). His‐tagged c‐Myc was purified from bacterial BL21 cells with Ni^+^‐NTA agarose (1018244, Qiagen) in accordance with standard procedures. The plasmid used in the purification of recombinant c‐Myc protein was constructed by Tsingke Biotechnology Co., Ltd. (Beijing). The stock concentration of c‐Myc labelled with the NT‐647‐NHS dye was 2.08 mM. The concentration of the MDK stock solution was 11.7 mM. The c‐Myc stock solution was diluted with PBSP to 416 nM. The MDK stock solution was serially diluted with PBSP (1× PBS+0.1% Pluronic F‐127; 1:1). A total of 16 concentrations of MDK working solution were prepared, and 10 µL of each concentration was placed in a tube. Next, 10 µL of 416 nM c‐Myc was added to each tube from 16 to 1, followed by mixing using a pipette. Monolith NT.115 standard treated capillaries were immersed in tubes from 1 to 16 and positioned in slots 1–16 on the device tray prior to the measurement.

### 2.18 Protein–protein docking

The protein sequence and crystal structure information were downloaded from the Uniprot (https://www.uniprot.org/) database, and the protein crystal structure was acquired from the PDB database (https://www.rcsb.org/). The HEX8.0.0 protein–protein docking method was used for all docking calculations. First, two protein structures were pretreated using the protein preparation module, and the pretreated protein structure was used as the initial structure of protein docking. During docking, the protein main chain remained fixed, and protein–protein docking was conducted. The results with the lowest interface scores were checked, and the docking results were analyzed using Pymol.

### 2.19 Small‐molecule‐protein docking

The configuration of the target protein was optimized using Maestro11 “Protein Preparation Wizard” tool, such as hydrogenation and water removal, and the potential active site of the target protein was forecasted using Sitemap software. The active sites that were forecasted were identified using the “Receptor Grid Generation” procedure. Using the “Ligand Docking” tool, the minimized target protein and its optimized primary ligand were molecularly docked. ACT001 interacted with different active sites on the target protein. The interactions between small molecules and target proteins were evaluated using the “Glide‐gscore” and “Glide‐energy” scores. Hydrogen bond, hydrophobicity, van der Waals force, and other interactions were considered by the function. The stability of the docking complex between small molecules and target proteins, as well as the matching and binding effects, was enhanced after the absolute value increased. The interactions between ACT001 and a target protein and those between a primary ligand and a target protein were compared.

### 2.20 Immunohistochemistry

Paraffin sections were deparaffinized using xylene I and xylene II for 15 min. Next, the sections were immersed in 100% alcohol I, 100% alcohol II, 95% alcohol, and 80% alcohol for 5 min in sequence. Then, the sections were rinsed with tap water for 5 min, followed by distilled water for 3 min and PBS for 3 min. This process was repeated three times. The sections were immersed in preheated citric acid buffer, heated in a microwave oven on medium heat for 3 min, and then cooled at room temperature for 5 min. The cycle of heating and cooling was performed three times. Finally, the sections were cleaned with PBST for 5 min three times. The sections were treated with 3% H_2_O_2_, left at room temperature for 10 min, and then rinsed with PBS for 3 min. This procedure was repeated three times. The section was sealed with goat serum for 30 min and then incubated overnight at 4°C with primary antibodies against MDK (11009‐1‐AP, Proteintech), c‐Myc (67447‐1, Proteintech), cyclin D1 (AF0931, Affinity), β‐catenin (AF6266, Affinity), E‐cadherin (AF0131, Affinity), N‐cadherin (AF5239, Affinity), vimentin (AF7013, Affinity), MMP‐2 (AF5330, Affinity), and MMP‐9 (AF5228, Affinity). After being washed with PBS, the sections were then incubated with the appropriate secondary antibodies for 30 min. Finally, DAB was used to enhance the colour, whereas hematoxylin was utilized for nucleus re‐staining. The slices were dehydrated to wax and sealed with neutral balsam.

### 2.21 Immunofluorescence staining

The cells were seeded into a confocal dish the day before, and the culture medium was removed once the cells reached a density of 30%–70%. After rinsing with PBS, the cells were treated with 4% paraformaldehyde at room temperature for 15 min. The cells were treated with 0.5% Triton X‐100 solutions for 15 min and washed with PBS. Then, 5% BSA was applied to the seal at room temperature for 30 min. The primary antibody targeting MDK and c‐Myc was applied and left to incubate at 4°C overnight, followed by washing with PBS. The secondary Alexa fluor 555‐labeled donkey antirabbit (A0453, Beyotime) and Alexa fluor 488‐labeled goat antimouse IgG (A0428, Beyotime) were added, left at room temperature for 1 h in the dark, and then rinsed with PBS. An antifading mounting medium with DAPI (P0131, Beyotime) was added, left at room temperature for 15 min, and then rinsed with PBS. It was observed under a confocal microscope. After dewaxing and rehydration, antigen repair, sealing, primary and secondary antibody incubation, and the addition of an antifading mounting medium with DAPI were performed, and the sections were photographed.

### 2.22 Ubiquitination stability detection

After the cells were treated, 10 µM MG132 (T2154, TargetMol), which is a proteasome inhibitor, was added, and the cells were harvested 4 h later. RIPA lysate with protease inhibitor was added to the cells, and the cells were lysed with an ultrasonic cell crusher at 60 W for 3 min. Then, co‐immunocoprecipitation (co‐IP) was performed using c‐Myc antibodies and protein A/G.

### 2.23 Proximity ligation assay assay

Proximity ligation assay (PLA) was performed using the Duolink In Situ PLA kit (Sigma‐Aldrich) in accordance with the manufacturer's instructions. Glioma cells (SF126, U118MG, and U251) were seeded on confocal dishes. Cells were fixed with 4% paraformaldehyde, permeabilized with 1% Triton X‐100, and blocked with 5% BSA in PBS for 30 min. Subsequently, the cells were incubated overnight at 4°C with primary antibodies against MDK (11009‐1‐AP, Proteintech, 1:100) and c‐Myc (67447‐1, Proteintech, 1:100). After washing three times with Wash Buffer A, the cells were incubated with PLA PLUS and MINUS probes for 1 h at 37°C. Ligation was performed at 37°C for 30 min, followed by amplification at 37°C for 100 min in accordance with the manufacturer's protocol. The slides were washed and mounted with a DAPI‐containing mounting medium (P0131, Beyotime). PLA signals, which were visualized as discrete fluorescent dots, were imaged using a confocal microscope (Nikon).

### 2.24 Statistical analysis

The expression levels of MDK mRNA in patients with different clinical and pathological characteristics were examined using a normal distribution test. Data following normal distribution were presented as mean ± SD and statistical analysis was performed using an independent sample *t*‐test, one‐way analysis of variance, and LSD‐t two‐comparison method. The relationship between MDK mRNA expression and survival time in patients with different types of brain glioma was analyzed using the Kaplan–Meier survival curve and log‐rank test. All statistical tests were bilateral, and *p* < .05 was considered statistically significant.

## 3 RESULTS

### 3.1 MDK is highly expressed in glioma and is associated with poor prognosis

**FIGURE 1:**
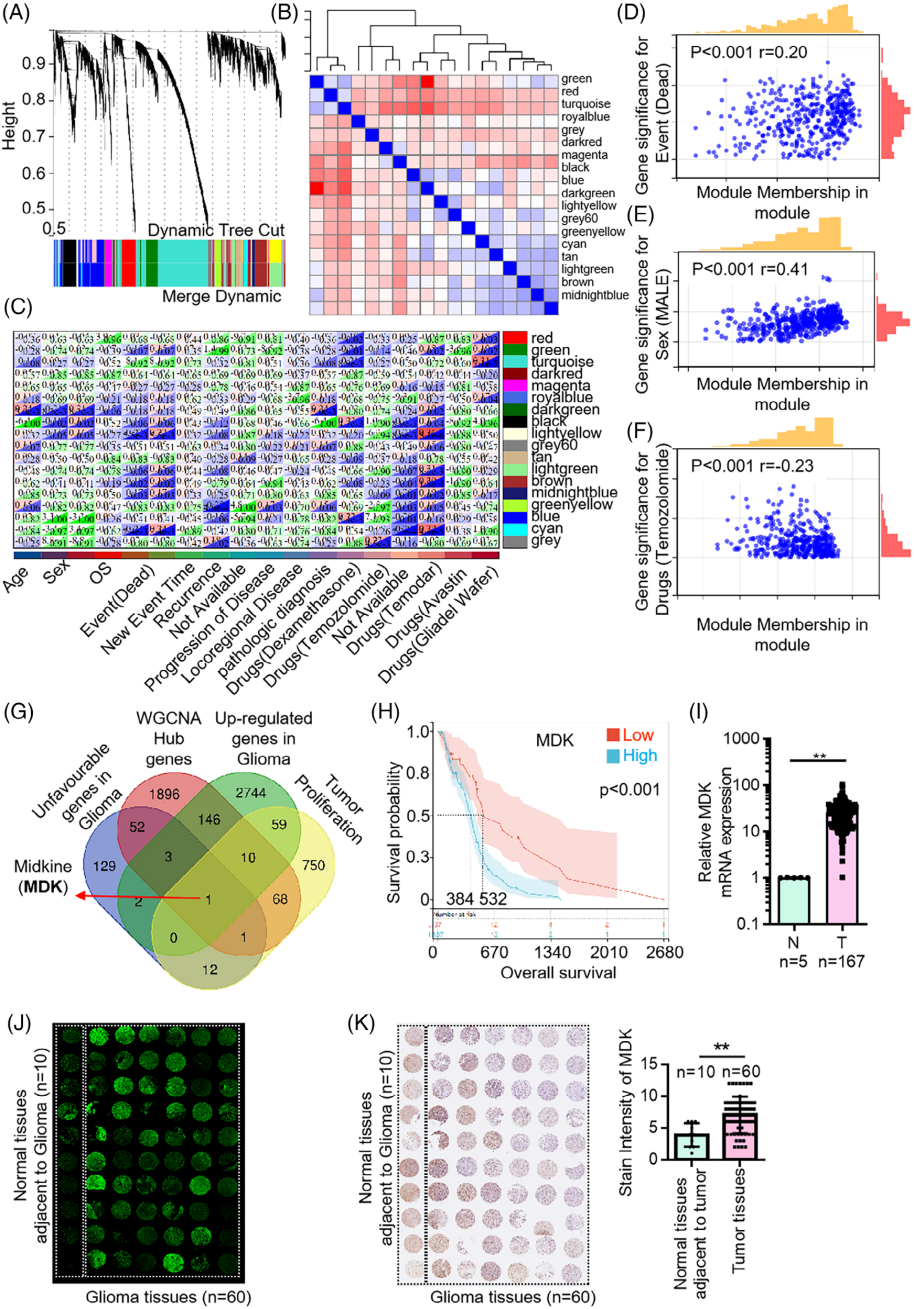
MDK is highly expressed in gliomas and is associated with poor prognosis. (A) Cluster dendrogram is a tree for hierarchical clustering. Based on the optimal soft threshold and expression profile data. The raw data are shown in Table S1. (B) Dule‐trait relationship is an association map between modules and phenotypic characteristics. The horizontal coordinate is the trait. The ordinate is the corresponding module, which is represented by the eigengene of each module. (C) Phenotype and module correlation diagram, where it can be determined whether the relationship between the module and the phenotype of concern is significant. (D) WGCNA analysis shows the correlation between module membership in the module and the survival status of patients with brain glioma. (E) WGCNA analysis shows the correlation between module membership in the module and the genders of the patients. (F) WGCNA analysis shows the correlation between module membership in module and drug resistance in the patients. (G) VENN analysis confirms the importance of MDK. Unfavourable genes in glioma (Table S2): refer to poor prognosis genes in glioma; WGCNA Hub genes (Table S3): The key genes of brain gliomas obtained through WGCNA analysis are highly correlated with the occurrence and development of brain gliomas; upregulated genes in glioma (Table 4): upregulated genes in glioma were obtained by analyzing the expression profile data of glioma patients in the TCGA database; tumour proliferation (Table S5): it refers to the gene whose gene function is related to tumour proliferation through analysis of Uniprot database. H–I. Bioinformatics analysis of the MDK gene. Survival analysis (H), expression level (I). (J) Immunofluorescence staining analysis of MDK intensity in tumour and adjacent tissues from the patients. (K) Immunohistochemistry staining analysis of MDK intensity in the tumour and adjacent tissues of the patients. Error bars represent mean ± SD, *p < .05, **p < .01.

To identify the key genes affecting brain glioma, WGCNA was performed on the sequencing expression data of patients with glioma. Data from 156 patients with different types of glioma were downloaded from the TCGA database (Table S1). A soft threshold was selected in accordance with the distribution of sequencing data shown in Figure 1A. This step ensured that the gene regulatory relationship conformed to a scale‐free distribution. A module clustering dendrogram was constructed in accordance with the co‐expression network, and 18 modules were identified. The grey module represented the genes that were not clustered (Figure 1B). The correlation coefficients and *P* values between each module and sample characteristics were calculated, and a module–sample trait association plot was generated (Figure 1C). Modules that exhibited a notable association (*p* < .05 and correlation coefficient of >.2) with glioma occurrence were selected for in‐depth analysis. WGCNA showed that module membership in the module was associated with patients’ survival status, gender, and drug resistance (Figure 1D–F). Unfavourable genes in glioma, WGCNA Hub genes, upregulated genes in glioma, and genes associated with tumour proliferation were used to narrow down the range of glioma‐related genes through VENN analysis. VENN analysis confirmed the importance of MDK (Figure 1G). After conducting Kaplan–Meier plotter analysis, the patients with glioma were categorized into the high‐ and low‐expression groups based on the median MDK expression level. The results indicated that patients with high MDK levels had a significantly lower OS compared with those with low MDK levels (*p* < .001; Figure 1H). Analysis of a large number of different glioma cases in the database showed that MDK was significantly upregulated in glioma cases than in the control tissues (Figure 1I). Furthermore, 10 adjacent non‐tumour tissues and 60 tissues were collected from patients with glioma. Immunofluorescence and immunohistochemistry findings indicated a notable increase in MDK expression in glioma tumour tissues compared with adjacent non‐tumour tissues (Figure 1J–K).

### 3.2 MDK promotes the growth and migration of glioma cells in vitro and in vivo

**FIGURE 2:**
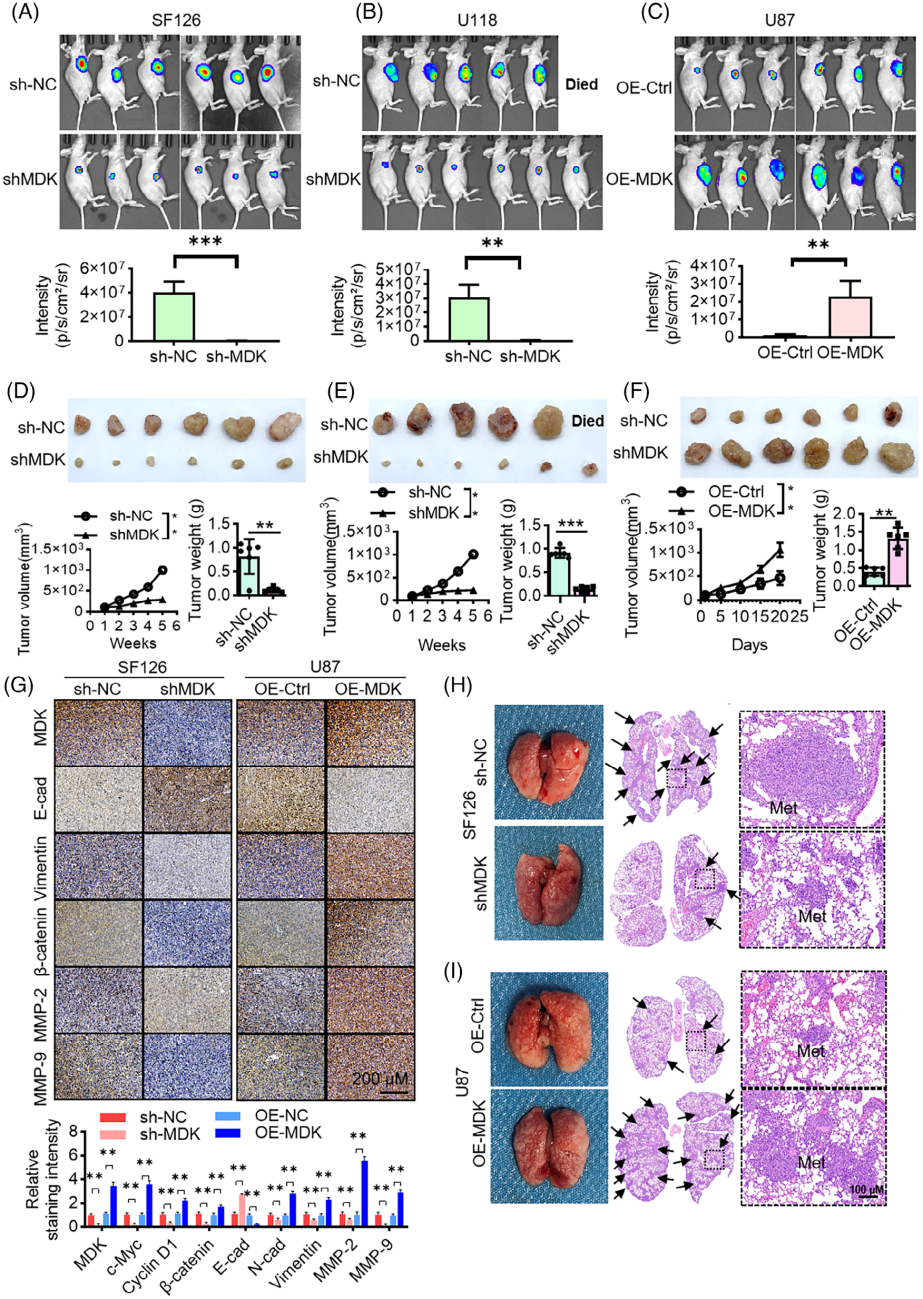
MDK promotes the in vivo growth of subcutaneous xenografted glioma tumours and lung metastasis. (A–C) Luciferase imaging and photon statistics of xenograft subcutaneous tumour in mice. (A, B) Knockdown of MDK inhibited the growth of the subcutaneous xenografts of the SF126 and U118MG cell lines. (C) Overexpression of MDK can promote the growth of subcutaneous xenograft tumours in U87 cells. (D–F) Tumour and tumour volume and weight statistics of nude mice. (G) The expression levels of E‐cadherin, vimentin, β‐catenin, MMP‐2, and MMP‐9 in tumour tissue were detected by immunohistochemistry. (H) Knocking down MDK can inhibit the formation of SF126 xenograft lung metastases in nude mice. (I) Overexpression of MDK can promote the formation of U87 xenograft lung metastases in nude mice. Error bars represent mean ± SD, *p < .05, **p < .01, ***p < .01.

First, the expression of MDK at the protein level was detected in multiple glioma cell lines, including U118MG, BT325, U251, SF126, SHG44, U87, and the normal glial cell line HEB. Based on the qRT‐PCR and Western blot results, the expression level of MDK was high in U118MG, U251, and SF126 cell lines and low in BT325, SHG44, and U87 cell lines (Figure S1A,B). To investigate the function of MDK, we first constructed cell lines with low MDK expression levels and overexpressed MDK. SF126, U118MG, and U251 cell lines, which exhibited relatively high MDK expression levels, were selected for the establishment of cell lines with MDK expression levels reduced by the sh‐MDK lentivirus. Similarly, stable cell lines with overexpressed MDK were established in U87, SHG44, and BT325 cell lines. The transfection effect is shown in Figure S1C,D. CCK8 assay (Figure S1E,F), EdU assay (Figure S1G,H), and clonal formation experiment (Figure S1K,L) indicated that reducing MDK levels suppressed the proliferation of glioma cells while increasing MDK levels enhanced their proliferation. Furthermore, after MDK was knocked down, the invasion of glioma cells in vitro was suppressed (Figures S1I,J). To verify the effect of MDK on metastasis and growth, an in vivo tumour formation experiment was conducted on 6‐week‐old male BALB/c nude mice. The cells were inoculated subcutaneously into the mice. Tumour formation was observed after 1 week. The findings indicated that knocking down MDK had a notable hindering impact on the development of tumours in glioma cells in vivo (Figure 2A,B,D,E). In the U87 cell line, the tumour volume of the OE‐MDK group significantly increased compared with the control group (*p* < .01). At the endpoint of the animal experiment, the tumour weight measured 0.408 ± .113 g for the control group and 1.323 ± .295 g for the OE‐MDK group. The tumour volume significantly increased in the OE‐MDK group in comparison with the control group (*p* < .01; Figure 2C,F). Tumour migration, invasion, and proliferation were significantly associated with E‐cadherin, vimentin, β‐catenin, MMP‐2, and MMP‐9. Therefore, studying the changes in the expression of these proteins is essential to investigate the effect of MDK on tumour progression. Immunohistochemical analysis revealed that suppressing MDK led to decreased levels of Vimentin, β‐catenin, MMP‐2, and MMP‐9. Nevertheless, the levels of E‐cadherin expression increased. The overexpression of MDK enhanced the levels of Vimentin, β‐catenin, MMP‐2, and MMP‐9. Nevertheless, E‐cadherin expression was inhibited (Figure 2G). In addition, the effect of MDK on lung metastasis was evaluated by intravenous injection of 100 µL of 2 × 10^6^ tumour cells. The mice were observed 28 days after intravenous injection of tumour cells. The mice in the SF126 sh‐NC group had visible lung deformities, increased volumes, and grey–white nodules of varying sizes on the surfaces. The nodules had a solid texture. Histological examination revealed a high number of tumour nodules, varying sizes of tumour cells with irregular nuclei, and basophilic cytoplasm in the tumour group. However, the lung tumour nodules were greatly reduced after MDK silencing (Figure 2H). In the U87 cell line, MDK overexpression promoted the number of lung tumour nodules (Figure 2I). Furthermore, HEB cells, which are normal glial cells, were selected to analyze the effect of MDK overexpression on normal cells. The proliferation capacity of HEB cells overexpressing MDK was evaluated using the EdU method. The clonogenic ability of the HEB cells overexpressing MDK was evaluated by a colony formation assay. The overexpression of MDK promoted the proliferation capacity of normal glial cells (Figures S1M, S2N). Similarly, cell invasion capacity was evaluated in HEB cells overexpressing MDK. The findings indicated that the overexpression of MDK enhanced the ability of noncancerous glial cells to invade (Figure S1O).

### 3.3 MDK interacts with c‐Myc, and a co‐expression correlation is observed between them

**FIGURE 3:**
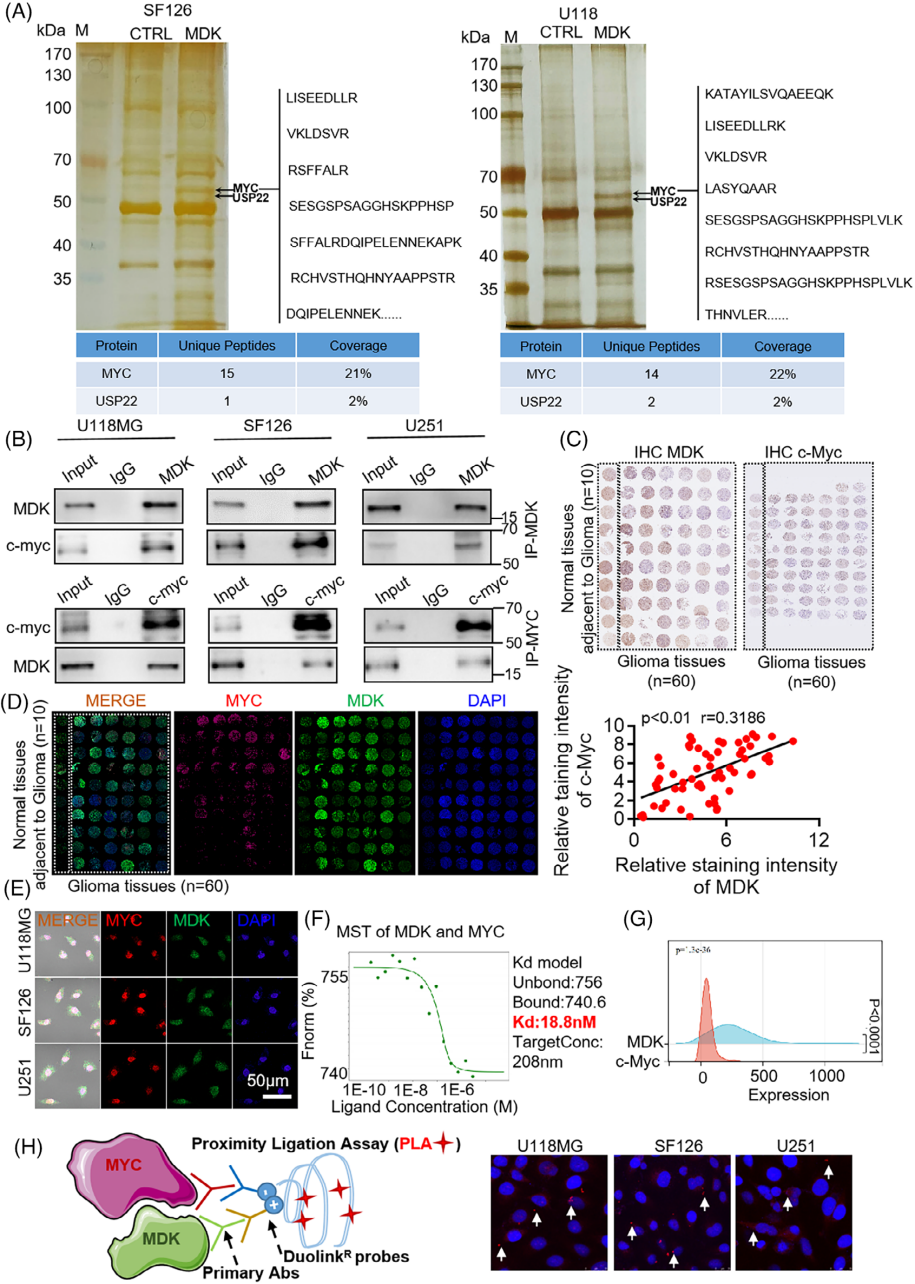
MDK directly interacted with c‐Myc and coexpressed with c‐Myc. (A) Total protein from U118MG and SF126 stably expressing FLAG‐MDK cells were immunopurified with anti‐FLAG beads. The eluents on the beads were separated by the SDS‐PAGE gel, silver dyeing was performed, and differential bands were cut and sent for mass spectrometry detection. The representative peptides and coverage of c‐Myc are shown. Additional detailed results are available in Table 6 and Table S7. (B) Cell lysates from U118MG, SF126, and U251 cells overexpressing MDK were immunoprecipitated and immunoblotted with antibodies against the indicated proteins. (C) Immunohistochemistry of MDK and c‐Myc in normal paracancer and glioma tissues. (D–E) Fluorescent staining analysis of MDK and c‐Myc interactions in glioma tissue and its adjacent tissues (D) and glioma cells (E). (F) MST (microscale thermophoresis) analysis of the interaction between MDK and c‐Myc. (G) Coexpression and peak plot analysis of MDK and MYC in the TCGA database. (H) Proximity ligation assay (PLA) demonstrated the endogenous interaction between MDK and c‐Myc. Error bars represent mean ± SD, *p <.05, **p < .01.

To further study the mechanism of MDK on glioma, a pull‐down experiment was conducted to identify MDK‐interacting proteins. After silver staining, the pull‐down results showed a differential band (50–70 kDa) between the MDK antibody incubation and control groups in SF126 and U118MG cells. Then, the bands were cut; enzymolysis was performed, and the proteins in the bands were identified by protein mass spectrometry. On the basis of the relative molecular weight and protein score identified by mass spectrometry, proteins with a relative molecular weight of 60 kDa and high mass spectrometry scores were further analyzed. Among them, 15 and 14 peptide fragments of c‐Myc were found in SF126 and U118MG cells, respectively. The protein coverage was 21% and 22%, respectively (Figure 3A). The results were reliable. Furthermore, a thorough examination of mass spectrometry data revealed the presence of USP22 in the MDK antibody treatment group compared with the control group in SF126 and U118MG cell lines. Subsequently, the interaction between MDK and c‐Myc was validated through immunoprecipitation. The total cellular proteins were extracted, immunoprecipitated with MDK and c‐Myc antibodies, and detected by Western blot. No MDK and c‐Myc bands were detected in the IgG group, whereas the bands of MDK and c‐Myc proteins were detected in the immunoprecipitation group (Figure 3B). The positive results of the co‐IP experiment were not due to nonspecific reactions of IgG. In addition, the interaction between MDK and c‐Myc was confirmed in the U87 cell line with low *MDK* gene expression investigated using co‐IP experiments. When MDK was overexpressed in the U87 cells, immunoprecipitation was performed with MDK and c‐Myc, followed by immunoblotting with corresponding antibodies. c‐Myc and MDK were harvested from U87 cells overexpressing MDK (Figure S2A). However, when U87 cells were not overexpressed with MDK, c‐Myc was not detected (Figure S2B). The above‐mentioned results indicated an interaction between MDK and c‐Myc. The immunohistochemistry and confocal microscopy observations of MDK and c‐Myc revealed that they were partially localized in the cytoplasm and had a degree of subcellular localization consistency (Figure 3C–E; Figure S2C). Molecular interaction techniques explored the mechanisms underlying molecular interactions by detecting the characteristics of protein–protein interactions and the interactions of other substances, particularly affinity constants. Microscale thermophoresis (MST) is widely used for the quantitative detection of molecular interactions. In this study, MDK and c‐Myc proteins were subjected to MST analysis, which showed a Kd value of 18.8 nM, indicating a specific protein interaction (Figure 3F). Co‐expression analysis of TCGA glioma sequencing data showed a co‐expression trend between MDK and c‐Myc expression levels in glioma tissues (Figure 3G). The PLA results demonstrate distinct punctate signals indicating the physical proximity of endogenous MDK and c‐Myc proteins within glioma cells. This result provides more compelling evidence for their direct interaction (Figure 3H). All the above‐mentioned experiments indicated an interaction between MDK and c‐Myc.

### 3.4 MDK impacts the ubiquitination of c‐Myc and the signalling pathway of Wnt/β‐catenin

**FIGURE 4:**
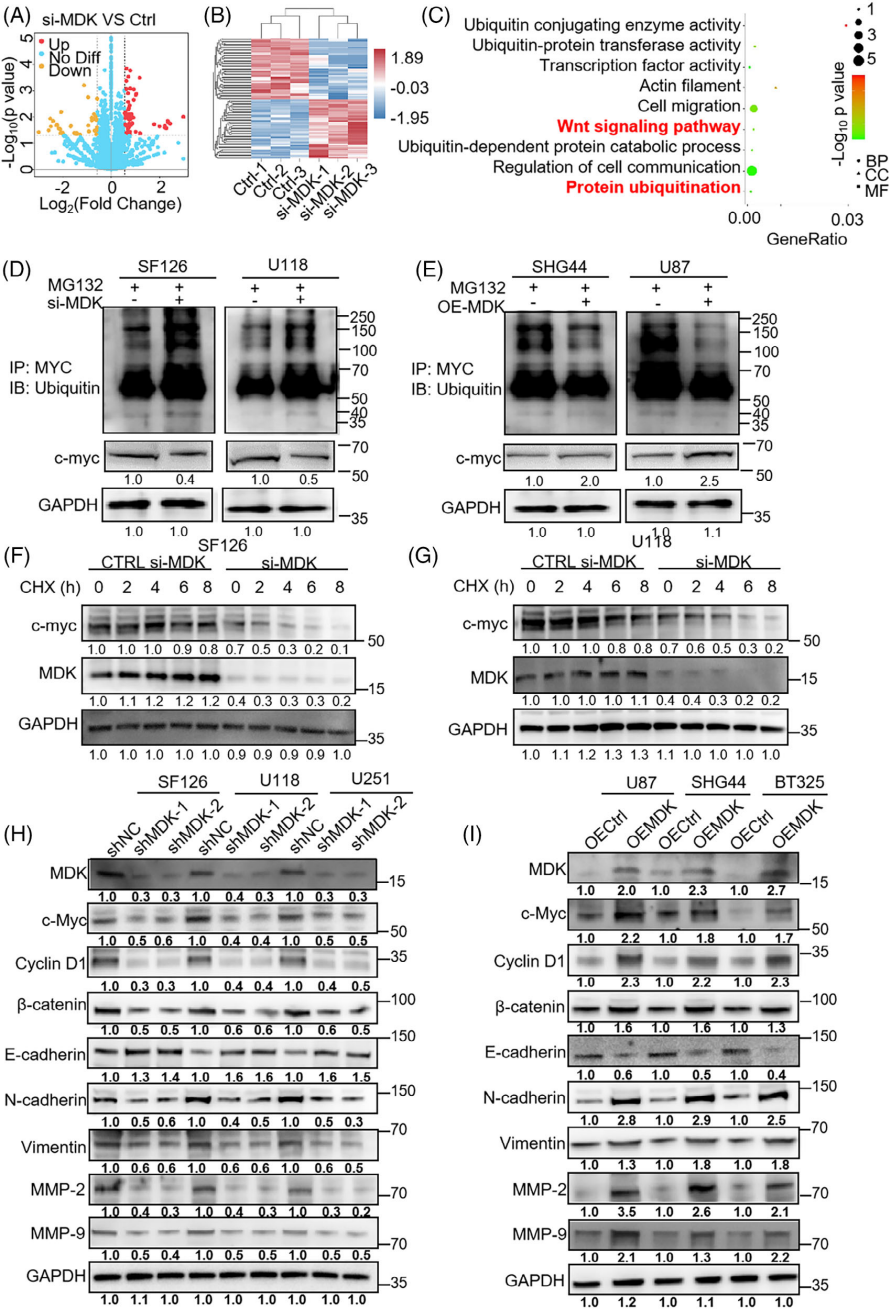
MDK affects the ubiquitination modification of c‐Myc and the Wnt/β‐catenin signalling pathway. (A–C) Results from proteomic analysis indicated that MDK affected the Wnt/β‐catenin signalling pathway and protein ubiquitination. The raw data are shown in Table S8. (A) Volcano plot analysis results showed differentially expressed proteins (Table S9). The red dots represent upregulated expression in the siMDK group compared with the control group, and the yellow dots represent downregulated expression in the siMDK group compared with the control group. (B) Cluster analysis results showed similarities and differences among the groups (control vs. siMDK) and the stability of the mass spectrometry results of the three repeated experiments (Table S10). (C) KEGG pathway enrichment analysis revealed the signalling pathway affected by MDK knockdown (Table S11). (D) After treatment with 10 mM MG132 for 4 h, siMDK‐U118MG and siMDK‐SF126 cell lysates were immunoprecipitated with c‐Myc and immunoblotted with ubiquitination antibodies. (E) After treatment with 10 mM MG132 for 4 h, OE‐MDK‐SHG44 and OE‐MDK‐U87 cell lysates were immunoprecipitated with c‐Myc and immunoblotted with ubiquitination antibodies. (F–G) 50 mg/mL cycloheximide was added (at different time points: 0, 2, 4, 6, and 8 h) to glioma cells transfected with siRNA to block protein synthesis. The expression of c‐Myc was then detected by Western blot. (H) MDK knockdown was performed on SF126, U118MG, and U251, and then a Western blot was used to detect the Wnt/β‐catenin signalling pathway and EMT pathway markers. (I) MDK was overexpressed in U87, BT325, and SHG44, and Western blot was used to detect the Wnt/β‐catenin signalling pathway and EMT pathway markers.

Proteomics provides comprehensive protein information in cancer cells, aiding in the understanding of the molecular processes involved in tumour formation. Hence, the impact of altered MDK levels on the protein expression profile in human glioma cells was investigated using proteomic analysis, leading to the identification of affected signalling pathways. Total proteins were extracted from MDK‐knockdown cells and wild‐type (WT) cells. After enzymolysis, mass spectrometry was utilized to identify proteins that were expressed differently. The mass spectrometry results are displayed in Table S8. The differentially expressed proteins were annotated and enriched using the GO database, and the involved signalling pathways were enriched using the KEGG database. In the MDK‐knockdown SF126 cells, a total of 151 proteins showed differential expression, with 69 being upregulated and 82 downregulated, thereby meeting the criteria of fold change ≥1.5 and *p* < .05 (Figure 4A,B). KEGG pathway enrichment analysis revealed that MDK knockdown mainly affected the Wnt signalling pathway and protein ubiquitination function, both of which are closely related to tumorigenesis (Figure 4C). However, whether MDK is involved in the regulation of the ubiquitination degradation of c‐Myc remains unclear. We hypothesized that MDK stabilizes the expression of c‐Myc by inhibiting its ubiquitination degradation. Following the addition of MG132, the decrease in MDK expression level in SF126 and U118MG cells led to the ubiquitination of c‐Myc protein, resulting in the enhanced degradation of c‐Myc (Figure 4D). Increased MDK levels in SHG44 and U87 cells suppressed the ubiquitination of the c‐Myc protein, leading to the enhanced stability of c‐Myc (Figure 4E). Afterwards, 50 µg/mL cycloheximide was added to glioma cells that were transfected with siRNA at various time points (0, 2, 4, 6, and 8 h) to inhibit protein synthesis. The findings indicated that the half‐life of c‐Myc in glioma cells transfected with siRNA‐MDK was significantly shorter compared with the control group (Figure 4F,G). These results indicated that MDK is involved in the ubiquitination degradation of c‐Myc protein. Moreover, the co‐IP mass spectrometry results indicated that USP22 is involved in ubiquitination degradation. Afterwards, the impact of MDK on the Wnt/β‐catenin signalling pathway and EMT markers in cancer cells was confirmed using Western blot analysis. Silencing MDK in SF126, U118MG, and U251 cells resulted in the decreased expression level of c‐Myc, cyclin D1, and β‐catenin proteins, as well as the markers of mesenchymal transition, such as N‐cadherin, vimentin, MMP‐2, and MMP‐9. However, the epithelial marker E‐cadherin was upregulated after MDK silencing (Figure 4H). MDK overexpression in U87, SHG44, and BT325 cells increased the expression levels of c‐Myc, cyclin D1, and β‐catenin proteins as well as the markers associated with mesenchymal transition, including N‐cadherin, vimentin, MMP‐2, and MMP‐9. However, the epithelial marker E‐cadherin was downregulated after MDK overexpression (Figure 4I). Overall, our findings indicate that MDK influences the ubiquitination of c‐Myc and the Wnt/β‐catenin pathway, leading to alterations in cell migration.

### 3.5 MDK affects the Wnt signaling pathway through c‐Myc

**FIGURE 5:**
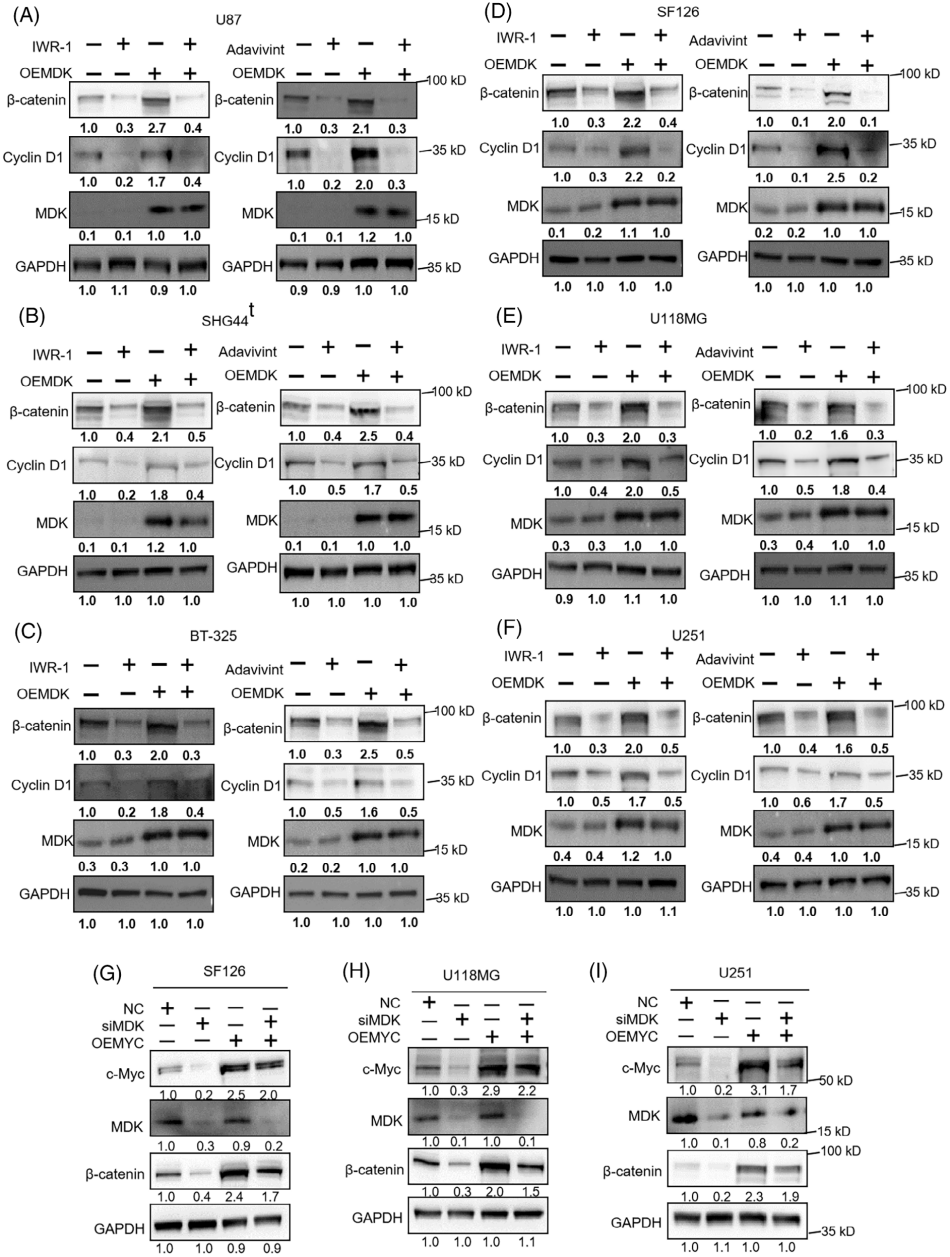
Inhibition of Wnt blocks the tumorigenic activity of MDK in glioma. U87, SHG44, BT‐325, SF126, U118MG and U251 cells were transfected with MDK‐overexpression plasmids and then treated with IWR‐1 (10 µM) or Adavivint (100 nM) for 24 h. The expression of β‐catenin, cyclin D1, MDK U87 (A), SHG44 (B), BT‐325 (C), SF126 (D), U118MG (E) and U251 (F) were then detected by western blot. (G–I) The rescue experiments were performed by overexpressing c‐Myc in MDK‐knockdown cells. 60 h after transfection, western blot was used to detect the expression levels of c‐Myc, MDK and β‐catenin in SF126 (G), U118MG (H), and U251 (I). Error bars represent mean ± SD, *p < .05, **p < .01, ***p < .001.

In confirming the impact of MDK on the Wnt/β‐catenin pathway, two Wnt inhibitors were selected, IWR‐1 and adavivint. Then, the effects of blocking the Wnt pathway on the oncogenic effects of MDK were investigated. MDK‐overexpression plasmids were transfected into U87, SHG44, BT‐325, SF126, U118MG, and U251 cells, followed by treatment with IWR‐1 (10 µM) or adavivint (100 nM) for 24 h. Afterwards, CCK‐8, EdU, and Transwell chamber assays were conducted. The findings indicated that increased MDK expression enhanced the proliferation capacity of U87, SHG44, BT‐325, SF126, U118MG, and U251 cells in comparison with the control group. However, when treated with OE‐MDK + IWR‐1 or OE‐MDK + adavivint, the proliferation capacity of the cells was markedly reduced compared with the OE‐MDK group (Figure S3A–C). The EdU assay results were consistent with the CCK‐8 results (Figure S3D–F). The findings of the Transwell assay indicated that upregulating MDK enhanced the invasiveness of U87, SHG44, BT‐325, SF126, U118MG, and U251 cell lines in comparison with the control group. However, when treated with OE‐MDK + IWR‐1 or OE‐MDK + adavivint, the cells exhibited a considerable reduction in invasive capacity compared with the OE‐MDK group (Figure S3G–I). Western blot analysis revealed alterations in the levels of cyclin D1 and β‐catenin (Figure 5A–F). In the OE‐MDK group, the levels of cyclin D1 and β‐catenin expression were elevated compared with the control group. However, the expression levels of cyclin D1 and β‐catenin decreased in the OE‐MDK + IWR‐1 or OE‐MDK + adavivint group compared with the OE‐MDK group. The findings indicate that inhibiting the Wnt/β‐catenin pathway can decrease the oncogenic impacts of MDK in glioma. We further verified the role of c‐Myc in the MDK/Wnt pathway by rescue experiments. The rescue experiment was conducted using U118MG, U251, and SF126 cell lines (Figure 5G–I; Figure S3J–L). Rescue experiments were performed by overexpressing c‐Myc in MDK‐knockdown cells. A series of experiments showed that c‐Myc restoration partially restored the effects of MDK gene knockout on cell proliferation and invasion, indicating that MDK influences the malignant progression of glioma cells through the c‐Myc/Wnt pathway.

### 3.6 Downregulation of MDK sensitizes human glioma cells to TMZ treatment

**FIGURE 6:**
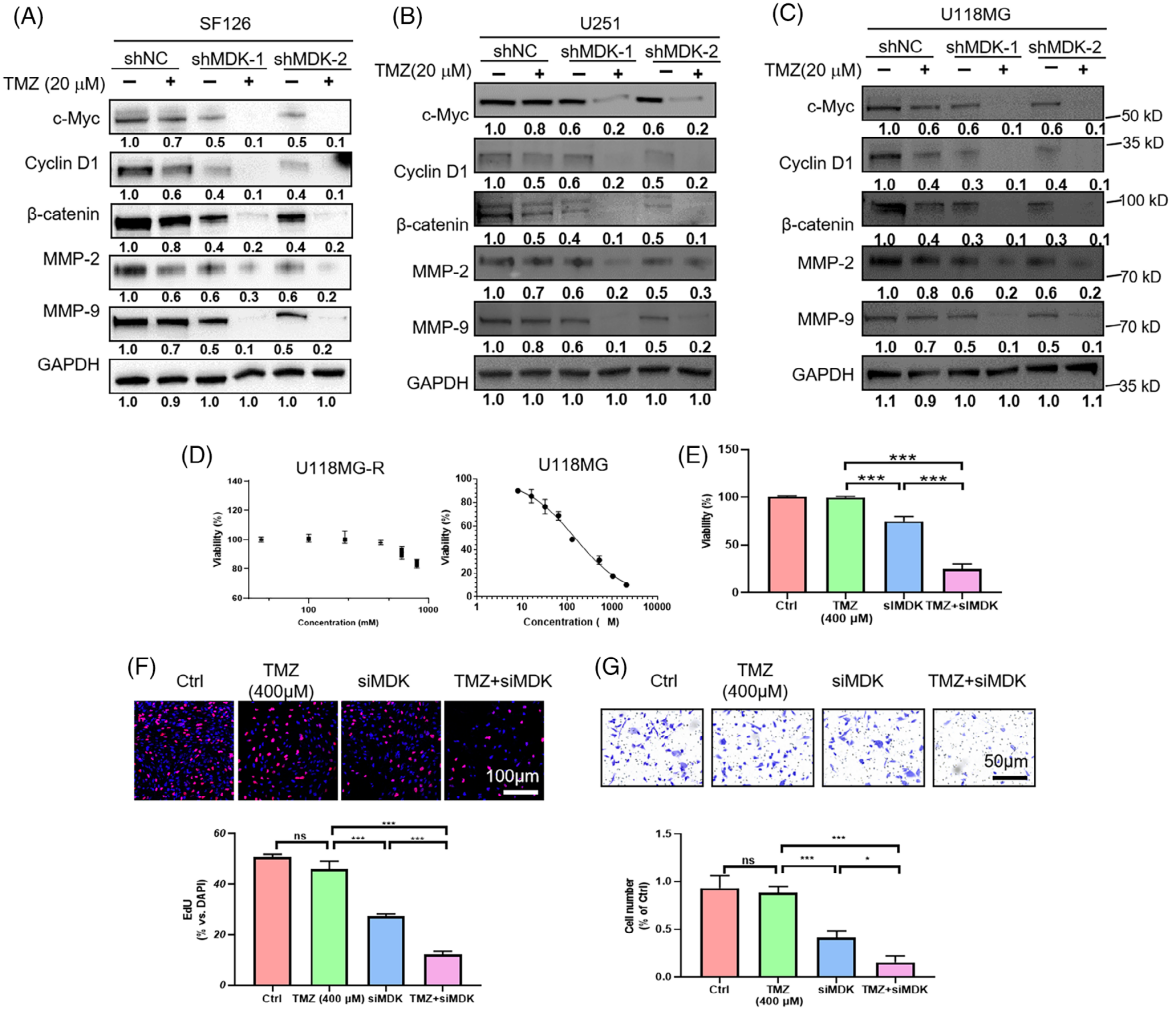
Downregulation of MDK enhances the sensitivity of human glioma cells to temozolomide treatment. (A–C) Western blot analysis of changes in the Wnt/β‐catenin signalling pathway in SF126 (A), U251 (B) and U118MG (C) cells treated under different conditions. (D, E) The survival rate of U118MG‐R and U118MG cell lines at different temozolomide concentrations was determined by CCK‐8 method. F. After MDK knockdown, the proliferation capacity of U118MG‐R cells after 400 µM TMZ treatment for 48 h was measured by the EdU method. (G) The effect of U118‐MG treated with 400 µM temozolomide and MDK knockdown on cell invasion was detected by the Transwell method. Error bars represent mean ± SD, *p < .05, **p < .01, ***p < .001.

In view of the above‐mentioned studies, MDK plays an essential role in glioma. MDK has been linked to drug resistance in several studies. This link has aroused our interest in the role of MDK in the treatment of glioma by TMZ. MDK knockdown was conducted on U118MG, SF126, and U251 cell lines, followed by the assessment of the impact of MDK expression levels on cell proliferation, invasion, and migration upon the addition of TMZ. In the shNC group, TMZ inhibited the proliferation of SF126, U118MG, and U251 cells in a dose‐dependent manner. However, the suppressive impact was restricted. In the shMDK group, even at a low dose, TMZ (20 µM) inhibited the proliferation of SF126, U118MG, and U251 cells (Figure S4A). The Transwell results indicated that in the absence of MDK knockdown, TMZ inhibited the invasive capacity of SF126, U118MG, and U251 cells in a dose‐dependent manner. After MDK knockdown, TMZ showed enhanced inhibitory effects on the invasive capacity of SF126, U118MG, and U251 cells (Figure S4B). The colony formation assay results indicated that MDK knockdown increased the inhibitory effect of TMZ on SF126 cell colony formation (Figure S4C). Western blot analysis indicated that reducing MDK levels suppressed the levels of c‐Myc, cyclin D1, β‐catenin, MMP‐2, and MMP‐9 in the SF126, U118MG, and U251 cell lines. In addition, in the group that received shMDK transfection and TMZ treatment concurrently for 72 h, the expression levels of c‐Myc, cyclin D1, β‐catenin, MMP‐2, and MMP‐9 protein were decreased (Figures 6A–C). In addition, the TMZ‐resistant cell line U118MG‐R constructed by our laboratory was used to verify the sensitization effect of knocking down MDK on TMZ. U118MG‐R cells exhibited negligible mortality after 72 h of treatment with 600 µM TMZ, whereas the IC_50_ value of the corresponding nonresistant cell line U118MG was 148 µM (Figure 6D). After 72 h of treatment with 400 µM TMZ, the U118MG‐R cells in the TMZ group exhibited minimal mortality, whereas the cells in the siMDK + TMZ group showed significantly increased cell death (Figure 6E). U118MG‐R cells Transwell and EdU assays confirmed that the sensitivity of U118MG‐R to TMZ increased after MDK was knocked down (Figure 6F,G).

### 3.7 ACT001 selectively inhibits the MDK/c‐Myc protein complex

**FIGURE 7:**
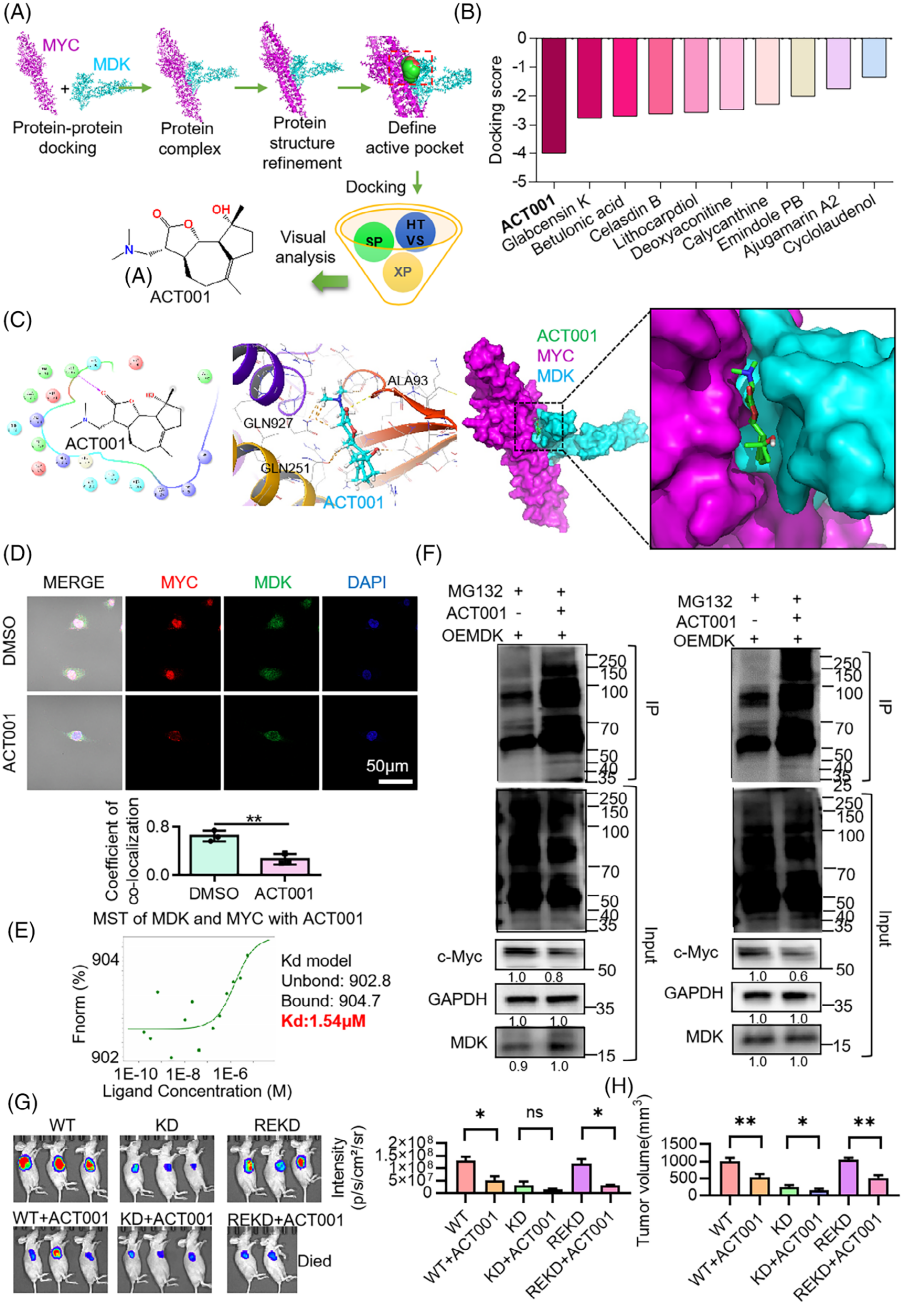
ACT001 targets and inhibits the MDK‐MYC protein complex. (A, B) High‐throughput drug screening based on the structure of the MDK/MYC protein complex: Through analysis of the structures of the MYC and MDK proteins and protein–protein docking using HEX software, the structure of the MDK/MYC protein complex was obtained. High‐throughput drug screening was then conducted based on this structure to identify compounds that could potentially inhibit the interaction between MDK and MYC, ultimately inhibiting cancer growth. (C) Structural display of the ACT001 and the protein complex: This image provides a detailed visualization of the interaction between the candidate drug ACT001 and the MDK/MYC protein complex. The three‐dimensional structure of the protein complex is shown, highlighting specific binding sites where ACT001 interacts with the complex (Ala93, Gln927, Gln251). (D) Immunofluorescence colocalization staining analysis of MDK and c‐Myc after treatment with 5 µM ACT001 for 48 h. E. After the addition of 1 µM ACT001, the dissociation constant (Kd) of MDK and c‐Myc was detected by the MST method. (F) The cells were incubated with 5 µM ACT001 or equivalent PBS for 72 h and treated with 10 µM MG132 for 4 h. After the cells were collected, they were immunoprecipitated using c‐Myc antibodies and immunoblotted with ubiquitination antibodies. (G) Mouse xenogenic subcutaneous tumour luciferase imaging representative images and photon statistics. (H) Statistical results of tumour volume in mice. Error bars represent mean ± SD, *p < .05, **p < .01.

Based on the results of previous experiments, docking analysis was performed between MDK and c‐Myc proteins, and then high‐throughput drug screening was conducted based on the structure of the MDK/c‐Myc protein complex. The screening results indicated that ACT001 had the highest docking score with the MDK/c‐Myc protein complex (Figure 7A,B). After molecular docking with the target proteins, ACT001 showed a docking score of −3.987 (Figure 7C). Docking analysis revealed the important role of hydrophobic interactions in this binding. ACT001 formed hydrogen bonds with the amino acid residue ALA93 and C–H bonds with GLN251, GLN927, and VAL102 in the protein complex. Immunofluorescence staining of MDK and c‐Myc in U118MG revealed a decrease in colocalization coefficient following ACT001 treatment in comparison with the control group (Figure 7D). The MST results demonstrated that ACT001 can dissociate the binding of MDK and c‐Myc, with a dissociation constant of 1.54 µM (Figure 7E). Our previous findings indicated that MDK can decrease the ubiquitination degradation of c‐Myc. To verify the functional inhibition of the protein complex by ACT001, MDK was overexpressed in the SF126 and U118MG cells and treated with ACT001. Then, the cells were treated with MG132 to inhibit proteasomal protein degradation. The results indicated that ACT001 treatment led to increased levels of c‐Myc ubiquitination in SF126 and U118MG cells. In addition, the ACT001‐treated group showed decreased expression levels of c‐Myc compared with the control group (Figure 7F). Ubiquitin contains seven lysine (Lys) residues that can be ubiquitinated. Among them, K48‐linked ubiquitination is associated with proteasomal degradation, while K63‐linked ubiquitination mainly participates in non‐degradative regulation. The experimental results showed that treatment with ACT001 led to a significant increase in K48‐linked ubiquitination, while the level of K63‐linked ubiquitination was reduced (Figure S5). These findings suggest that ACT001 promotes the activation of the proteasomal degradation pathway, as indicated by increased K48‐linked ubiquitin chains, while simultaneously suppressing non‐degradative signalling processes associated with K63‐linked ubiquitin chains. Considering previous co‐IP mass spectrometry results, we hypothesized that USP22 is involved in this ubiquitination degradation. The SF126 cell lines that knocked down MDK (known as KD) and returned MDK (known as REKD) were constructed. WT, KD, and REKD cells were planted under the skin of the mice. When the tumour volume was approximately 100 mm^3^, it was divided into six groups with six animals in each group, including the WT group, WT+ACT001 group, KD group, KD+ACT001 group, REKD group, and REKD+ACT001 group. A total of 200 mg/kg of ACT001 was orally administered six times a week for 5 weeks, and the other groups were given the same volume of solvent. The experimental results indicated that the tumour inhibition rate of the WT+ACT001 group was 47% compared with the WT group; the tumour inhibition rate of the KD+ACT001 group was 36% compared with the KD group, and the tumour inhibition rate of the REKD+ACT001 group was 51% compared with the REKD group. After knocking down MDK, the inhibitory effect of ACT001 on the tumour was decreased compared with the WT group (*p* < .05), but ACT001 still had an inhibitory effect. When MDK expression was restored, the inhibitory effect of ACT001 on tumours was comparable to that of the wild type (Figure 7G,H). The above‐mentioned experimental results indicate that ACT001 can exert anti‐tumour effects by targeting MDK, but ACT001 has other targets.

### 3.8 ACT001 inhibits glioma progression and enhances glioma sensitivity to TMZ

**FIGURE 8:**
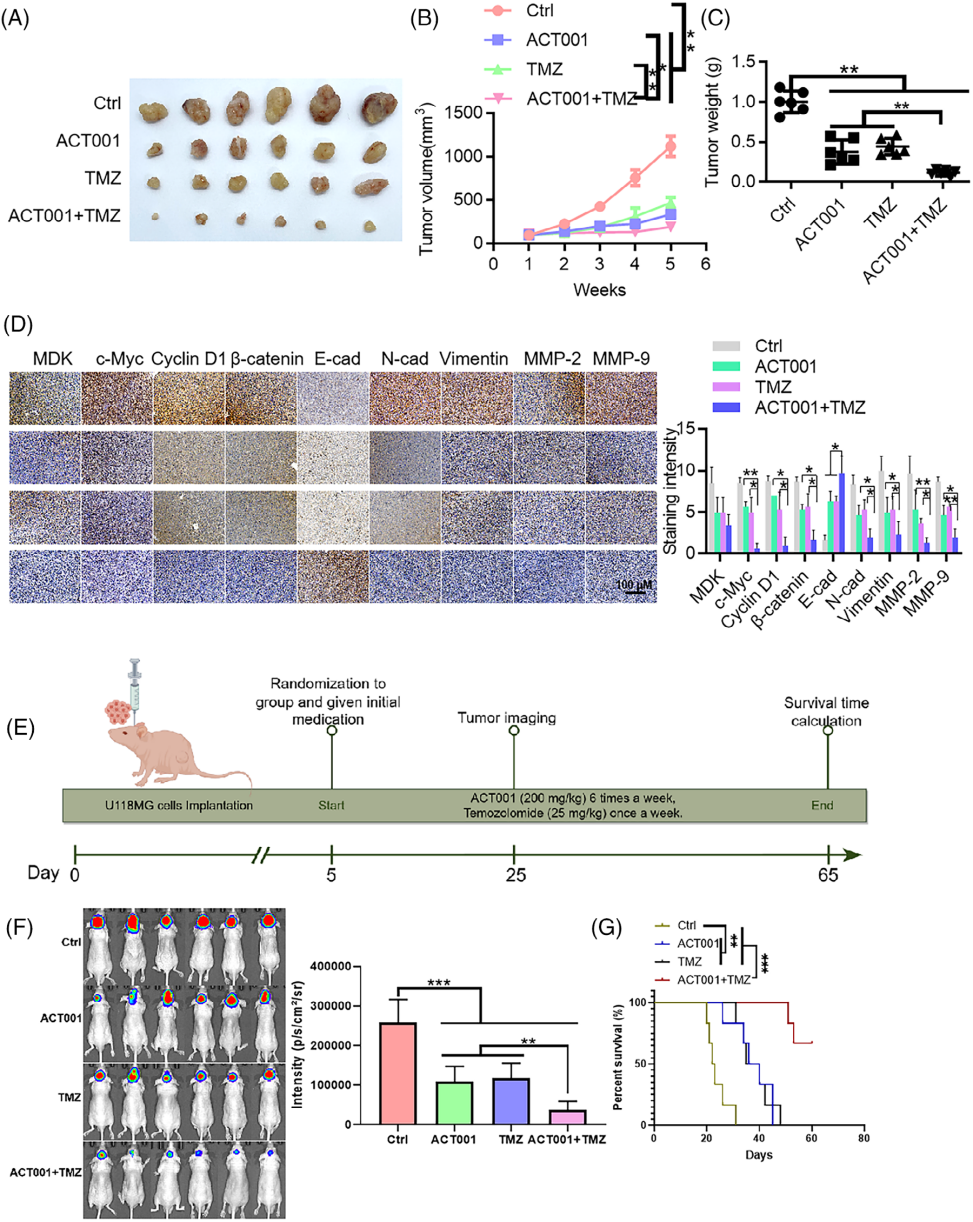
ACT001 inhibits glioma progression and increases glioma sensitivity to temozolomide. (A–D) The results of xenograft experiments in nude mice confirmed that ACT001 enhanced the anti‐tumour effect of temozolomide. (A) The tumour of CTRL, ACT001, temozolomide and ACT001+temozolomide groups. Statistical results of tumour volume (B) and tumour weight (C) in different groups of mice. (D) Immunohistochemical staining results and statistical results of the tumour tissues of mice in different administration groups. (E) Flowchart of in situ glioma model experiment in mice. (F) On the 20th day after grouping, the results of luciferase imaging and intensity statistics of mice in different groups were obtained. (G) Survival curve of mice with in situ glioma model. Error bars represent mean ± SD, *p < .05, **p < .01, ***p < .001.

**FIGURE 9:**
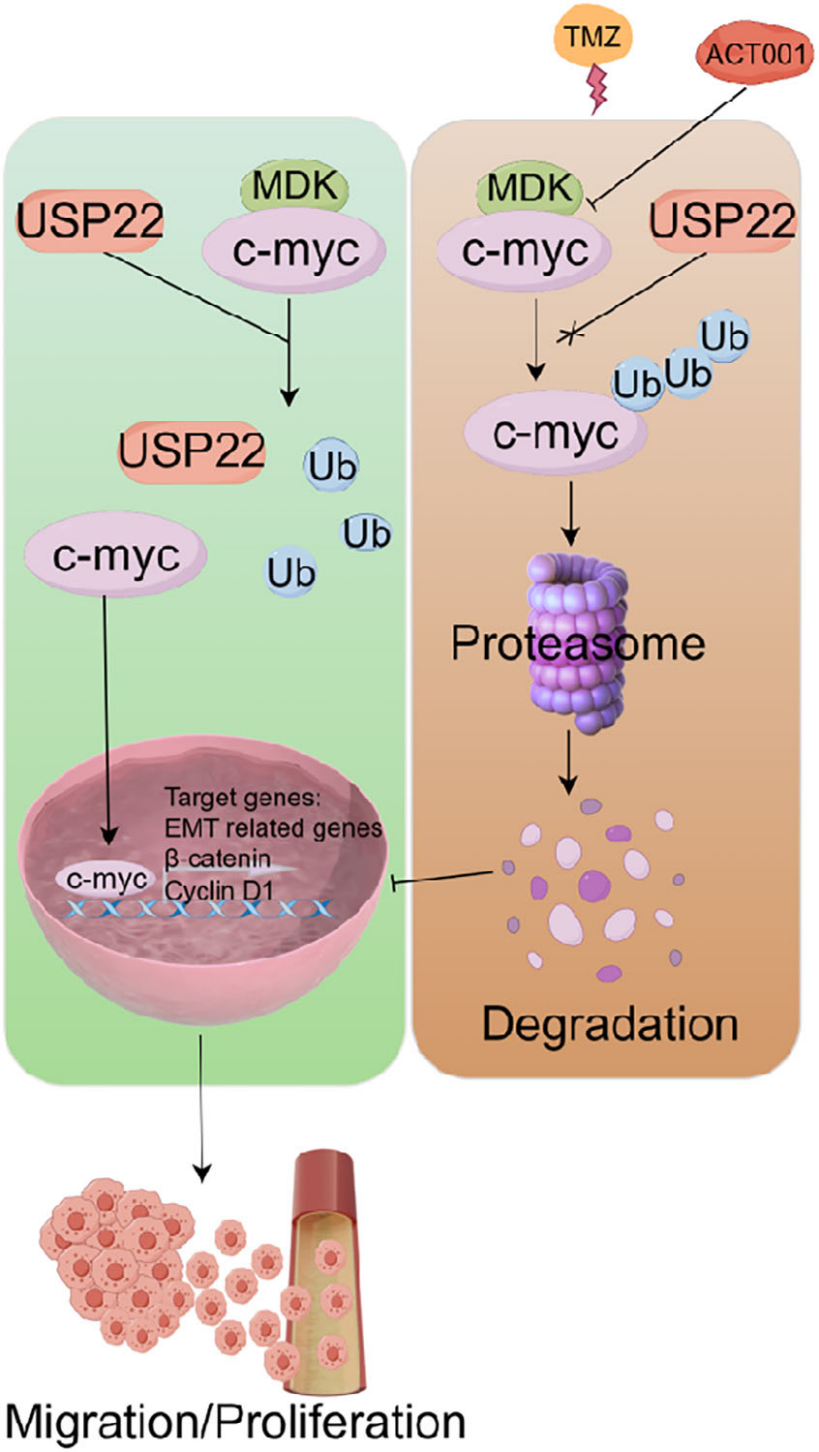
The mechanism of how the combination of ACT001 and temozolomide inhibits the progression of glioma was described.

ACT001 promoted the ubiquitination degradation of c‐Myc by targeting MDK, and MDK knockdown exerted a sensitization effect. ACT001 combined with TMZ is a promising strategy for glioma treatment. In the SF126, U118MG, and U251 cell lines, the ACT001‐treated group exhibited slower proliferation than the control group, and this effect showed a dose‐dependent relationship (Figure S6A). The Transwell assay results indicated that ACT001 inhibited the invasive capacity of the SF126, U118MG, and U251 cells in a dose‐dependent manner (Figure S6B). A colony formation assay was conducted to assess the mechanism by which the chemosensitivity of glioma cells was affected by the combination of ACT001 and TMZ. Under ACT001 treatment, SF126 and U118MG cells exhibited enhanced sensitivity to TMZ (Figure S6C), and the number of surviving cells was significantly low. Western blot analysis indicated that the combination treatment of ACT001 and TMZ led to decreased expression levels of c‐Myc, cyclin D1, β‐catenin, N‐cadherin, vimentin, MMP‐2, and MMP‐9 compared with TMZ treatment alone (Figure S6D). U118MG cells (2 × 10^6^) were inoculated in the right axilla of each nude mouse. Once the tumour grew to a certain size, the mice were divided into four groups consisting of six mice each group: control, ACT001, TMZ, and ACT001+TMZ groups. The results of the xenograft subcutaneous tumour experiment showed that the tumour volume of the control group increased gradually with treatment time. At the end of the experiment, the size of the tumour in the ACT001 + TMZ group was notably smaller than that in the control and single‐drug administration groups, with a statistical significance of *p* < .01 (Figure 8A–C). In the xenograft model, the tumour inhibition rates for the ACT001, TMZ, and ACT001 + TMZ groups were 76.59%, 63.74%, and 92.07%, respectively. In accordance with the Bliss model rules,^28^, ^32^ the combination index of ACT001 and TMZ is greater than 0 (CI = .006), indicating that the combination shows a synergistic effect on the xenograft model. Immunohistochemical findings indicated that the ACT001+TMZ treatment group had significantly reduced levels of c‐Myc, cyclin D1, β‐catenin, N‐cadherin, vimentin, and related matrix proteins such as MMP‐2 and MMP‐9 compared with the control group (Figure 8D). Furthermore, considering the presence of the blood–brain barrier, we performed a mouse brain orthotopic transplantation tumour study. The results indicated that the combination group had a significantly improved therapeutic effect and prolonged survival time of mice compared with the control and monotherapy groups (Figure 8E–G). In the in situ model, by day 48 of treatment, all animals in the monotherapy groups had died, whereas the survival rate for the ACT001 + TMZ group on day 60 of treatment was 66.7%, providing further evidence for the synergistic effect of the combination of ACT001 and TMZ. Therefore, ACT001 targets the MDK/c‐Myc complex to inhibit the malignant progression of glioma and increases its sensitivity when combined with TMZ (Figure 9).

## 4 DISCUSSION

Glioma is characterized by its tendency to recur and invade surrounding tissues.^33^ Given the unique location of its growth, surgical intervention and chemotherapy/radiotherapy have limited efficacy, making glioma a challenging disease to treat in the nervous system. The advancement of molecular biology has led to the utilization of different tumour biomarkers in the prevention and treatment of cancer.^34^

MDK is primarily expressed during embryonic development, whereas its expression levels in postnatal tissues are very low.^35^ At present, in adult organisms, MDK is only expressed in the kidney and intestinal epithelial tissues. However, in many pathological processes, including tissue ischemia,^36^ inflammation,^37^ and tumours,^11^ MDK expression is often increased, and high levels of MDK expression are often associated with disease progression. Our study found that among all patients with glioma or among glioma patients classified into high and low‐grade subtypes based on malignancy, those with high MDK mRNA expression had shorter survival and worse prognosis compared with those with low expression. Thus, expression of MDK mRNA could forecast the outcome for individuals with glioma, where a high expression level is linked to a negative prognosis.

Despite the success of advanced surgery and radiation treatments, the survival rate after 5 years for patients with advanced glioma is below 6%. Prior research has indicated that MDK is essential in the development and occurrence of tumours.^38^ The functional polymorphism of MDK has been linked to the risk of cancer and the clinical results in advanced colorectal cancer. Research has shown that MDK can control the proliferation and invasion of ectopic endometrial cells.^39^ Nevertheless, its expression in glioma tissues and the regulatory mechanism in glioma cells remain ambiguous. This study found an elevated expression level of MDK in human glioma tissues. In studying the role of MDK in the advancement of glioma, in vitro experiments were performed, which demonstrated that reducing MDK levels hindered the proliferation and migration of glioma cells. In addition, we discovered that reducing MDK expression increases responsiveness to chemotherapy, indicating that MDK could serve as a biomarker and treatment target for gliomas. In this research, plasmid transfection was utilized to increase the expression level of MDK in glioma cells, and stable cell lines that expressed MDK were selected through hygromycin screening. Using BALB/c nude mice, we established a glioma xenograft model and found that MDK overexpression significantly increased tumour volume and weight. Moreover, MDK overexpression promoted the formation of lung metastases, indicating that MDK can promote tumour growth in the xenograft model, which is consistent with previous reports. A correlation was also found between MDK expression and the levels of EMT markers and proteins related to the Wnt/β‐catenin pathway.

Our studies have found that the expression level of the MDK gene is significantly upregulated in glioma, confirming the correlation between MDK gene expression changes and glioma. The research demonstrated that MDK can control the Wnt/β‐catenin pathway in glioma cells by interacting with the oncogene c‐Myc. This interaction is essential for cell proliferation, differentiation, growth, and death, ultimately contributing to the initiation and progression of tumours. To clarify the relationship between MDK and c‐Myc and the regulation of their protein complex on the biological behaviours of glioma, we examined the expression of the MDK and *c‐Myc* genes in glioma tissues and adjacent tissues. The results showed that compared with adjacent tissues, MDK and c‐Myc were significantly upregulated in glioma tissues, and the expression of MDK and *c‐Myc* genes was associated with the occurrence of glioma. Additional statistical analysis revealed a positive correlation between the expression levels of MDK and c‐Myc in glioma tissues and adjacent tissues, indicating an inherent regulatory association between the two proteins. To further clarify the regulatory relationship between MDK and c‐Myc, we transfected MDK gene‐specific siRNA into glioma cells to silence the expression of the MDK protein. Western blot analysis confirmed a significant downregulation of c‐Myc protein. Therefore, a regulatory relationship can be found between MDK and c‐Myc in glioma cells, and changes in MDK expression can affect the expression and function of c‐Myc. Furthermore, multiple research studies have demonstrated the impact of USP22 on controlling the ubiquitination status of c‐Myc. Our study also identified the existence of USP22. Therefore, the interaction between MDK and c‐Myc is crucial for recruiting USP22 to deubiquitinate c‐Myc. In addition, E3 ubiquitin ligases play a crucial role in the progression of glioma by selectively mediating the ubiquitination of key tumour‐related proteins, thereby regulating their degradation, stability, and function.^40^ Such regulation affects a variety of malignant behaviours of glioma cells, including proliferation, migration, invasion, apoptosis, and drug resistance. Major families, such as Cullins and TRIMs, are involved in controlling central signalling pathways (e.g., cell cycle, MAPK/PI3K‐Akt) and in maintaining glioma stem cells. Cullin complexes target molecules such as Ras, NF1, and Bcl‐2, whereas TRIM proteins influence cell migration and immune responses through pathways like IQGAP1/CDC42 and STAT3. Moreover, E3 ubiquitin ligases are closely associated with therapeutic resistance and remodelling of the tumour microenvironment. In future studies, it will be of great significance to further investigate which specific E3 ubiquitin ligase is involved in the MDK/c‐Myc axis. Collectively, the MDK gene may influence cancer progression through its interaction with c‐Myc. Further studies will provide a new theoretical foundation for the study of the metastatic mechanism of glioma and a novel focus for treating glioma.

With the population ageing, the incidence of glioma further increases, which severely threatens human health.^41^, ^42^ The occurrence, development, migration, invasion, and recurrence of glioma involve changes in many genes and proteins. Molecular targeted therapy targeting key molecules in tumour occurrence, promoting tumour cell apoptosis, and inhibiting tumour formation has emerged as a popular area of focus in cancer treatment studies.^43^ Previous studies have shown that MDK is highly expressed in glioma cells. Consequently, focusing on MDK has emerged as a novel strategy for treating glioma. Moreover, the blood–brain barrier restricts the clinical application of many compounds that show effectiveness in laboratory settings. Several clinical studies have also verified that ACT001 can cross the blood–brain barrier and have an anti‐glioma therapeutic impact.^44^, ^45^, ^46^ Our previous preclinical studies have also shown that ACT001 can be enriched in the brain through the blood–brain barrier.^47^, ^48^ The study findings indicated that ACT001 effectively hinders the growth of glioma cells, with its effectiveness varying based on time and concentration. ACT001 markedly inhibits the clonogenic and migratory abilities of glioma cells. c‐Myc is an acknowledged oncogene that impacts cell proliferation, migration, and invasion by controlling downstream target gene expression. Excessive c‐Myc expression in oesophagal cancer tissues controls the occurrence and progression of cancerous cells and boosts their malignant behaviour, frequently resulting in poor prognosis in patients. We discovered and validated the interaction between MDK and c‐Myc and found that ACT001 can target the MDK/c‐Myc complex to inhibit tumour proliferation and growth.

The clinical application of TMZ represents a significant breakthrough in the chemotherapy of glioma.^49^ At present, TMZ‐based chemotherapy is commonly utilized for adjuvant treatment in high‐grade gliomas and salvage treatment in recurrent gliomas and low‐grade gliomas with poor prognostic indicators.^50^ Based on current evidence‐based medicine, chemotherapy plays an important role in the management of gliomas, although its effectiveness in clinical practice remains unclear.^51^ Despite the use of adjuvant TMZ chemotherapy as the current standard, the 5‐year survival rate for newly diagnosed gliomas remains at 9.8%.^52^ Therefore, exploring the mechanisms underlying drug resistance in gliomas and developing rational chemotherapy regimens are urgently necessary to improve treatment efficacy and prognosis.^53^ MDK is associated with the progression and drug resistance of many types of cancer,^22^ such as gastric cancer,^23,24^ biliary tract cancer,^25^ and glioma.^26, 27^ Xuehui Yu et al. found that MDK enhances p‐JNK expression through Notch1 and subsequently increases the expression levels of stemness markers (such as CD133 and Nanog), thereby promoting TMZ resistance.^28^ This study revealed that MDK impacted the Wnt signalling pathway by ubiquitinating c‐Myc, thereby influencing the resistance of glioma cells to TMZ. Notably, c‐Myc has a strong connection with the stemness and resistance to drugs in cancer cells.^29^ The abnormal regulation of MDK expression has been observed in brain tumours. A decrease in MDK levels in xenograft tumours in mice hinders tumour growth, prolongs the survival time of mice, and enhances tumour activity by various mechanisms, including the promotion of tumour cell growth and initiation of increased cell invasion. MDK interacts with c‐Myc, leading to the activation of the Wnt/β‐catenin signalling pathway. Based on these findings, this study demonstrated that silencing MDK reduces the resistance of glioma cells to TMZ treatment. The discovery of the MDK/c‐Myc axis provided novel therapeutic targets for drug resistance in gliomas.

Our previous studies confirmed that PAI1 is a target of ACT001.^28^ In addition, the active metabolite of ACT001, MCL, can covalently bind to pyruvate kinase M2 and inhibit tumour proliferation and occurrence.^54^ In addition, ACT001 can directly bind to IKKβ/STAT3 to induce apoptosis and inhibit the migration of glioma cells.^55^ These studies indicated that ACT001 plays an anti‐tumour therapeutic role through multiple targets.^56^ Consequently, further investigation of the mechanisms of ACT001 is crucial for clinical treatment, and it can be used in selecting the right chemotherapy drugs for combined therapy. Our research demonstrated that ACT001 decreased the proliferation of glioma cells and their resistance to chemotherapy through its impact on intracellular signal transduction. In addition to the mechanisms discussed above, recent studies have revealed that MDK can signal through the ALK receptor to sustain the self‐renewal and tumorigenic capacity of glioma‐initiating cells and that this pathway may contribute to temozolomide resistance.^17^ Moreover, a phase Ib clinical trial has evaluated the combination of the ALK inhibitor crizotinib with standard therapy in newly diagnosed glioblastoma patients, suggesting that targeting the MDK‐ALK axis may offer a new therapeutic strategy to overcome drug resistance.^57^ Our findings, focusing on the MDK/c‐Myc complex, are complementary to these reports and extend the potential spectrum of MDK as a therapeutic target in glioma. Future studies may benefit from integrating interventions targeting MDK/c‐Myc and MDK‐ALK axes for more effective reversal of therapy resistance. The intervention of ACT001 effectively suppressed the proliferation of glioma and tumour formation in nude mice while increasing glioma sensitivity to chemotherapy. Therefore, ACT001 is a potential agent for glioma treatment, providing good prospects for adjuvant glioma treatment. Despite being in the preliminary exploration stage, therapies targeting the MDK/c‐Myc complex have shown great application potential, indicating that MDK is a prognostic or diagnostic marker.

A recent small phase ІІ clinical study showed that therapies combining ACT001 and TMZ exert good therapeutic effects on patients with recurrent glioma multiforme, but the specific mechanism remains unclear.^44^ The findings of this study may provide a comprehensive understanding of the mechanisms underlying these effects and may contribute to the development of novel therapeutic targets for brain glioma. Treatments combining ACT001 and TMZ may be suitable for small communities and individuals with shared genetic factors.^58^ GBM cell lines are usually used in in vitro studies to determine the therapeutic effects of drugs, but the results of these experiments are inadequate to represent the results of actual clinical trials because of differences in many aspects of tumours in the human body and tumour cell lines, such as membrane similarities. Therefore, clinical studies are necessary to confirm the relationship between them.^59^ In addition, factors, such as whether a patient initially underwent radiation surgery, surgical resection, targeted therapy, or immunotherapy, should be considered in future studies. This approach may help to explore the feasibility of using ACT001 in combination with other treatments.^60^

In summary, existing literature indicates that MDK is widely regarded as an important biomarker in gliomas, with its overexpression closely associated with tumour proliferation, invasiveness, and chemoresistance. Yu et al.^15^ demonstrated that MDK promotes TMZ resistance in glioblastoma by enhancing cancer stem‐cell‐like characteristics. Glioma stem cells (GSCs) promote resistance to temozolomide (TMZ) in glioma by regulating cellular self‐renewal, suppressing apoptosis, and modulating critical signalling pathways.^61^ Furthermore, Han et al.^10^ found that inhibiting MDK can reduce the survival of glioblastoma tumour spheres by inducing cell cycle arrest and apoptosis. These studies provide a foundation for considering MDK as a potential therapeutic target. The novelty of the present study lies in our confirmation of the interaction between MDK and c‐Myc, as well as the elucidation of the role of the MDK/c‐Myc complex in TMZ resistance. By screening the small‐molecule inhibitor ACT001, the interaction between MDK and c‐Myc was successfully disrupted, thereby promoting apoptosis in TMZ‐resistant glioblastoma cells and inhibiting cell proliferation. This finding provides new insights and strategies for overcoming chemoresistance in gliomas. In addition, our research demonstrates that MDK influences tumour progression by regulating the Wnt/β‐catenin signalling pathway. This elucidation not only enriches the understanding of MDK's role in glioma biology but also identifies new potential targets for future clinical treatments. Therefore, MDK not only functions as an important biomarker but also serves as a crucial target in glioma therapy. Future studies could further explore the mechanism of the MDK/c‐Myc complex and the clinical potential of ACT001.

## 5 CONCLUSION

This study provided preliminary evidence of the role of the *MDK* gene in the development and progression of glioma, providing a theoretical basis for the identification of targets for gene therapy. At molecular, cellular, and animal levels, ACT001 alone and in combination with TMZ effectively inhibited MDK. Given that almost all patients with glioma eventually develop drug resistance, the discovery of MDK targets and the activity of ACT001 are of high clinical value. Moreover, MDK was found to be a potential biomarker for glioma, and targeting MDK may be beneficial for glioma treatment. Subsequent research can focus on screening patients with high MDK expression levels, as they may benefit from MDK inhibitors. The findings of this study may aid investigations on therapies targeting MDK and related biomarkers in glioma, the development of ACT001 as a glioma treatment, and the expansion of reference treatment protocols for glioma.

## AUTHOR CONTRIBUTIONS

Yaxin Lu, Weilong Zhong, Genbei Wang and Xiaonan Xi designed this study. Xiaonan Xi and Xiaojing Ding performed the study. Qianqian Wang and Ning Liu were involved in the collection and analysis of proteomic data. Bangmao Wang participated in the analysis of clinical data. Xiaonan Xi and Weilong Zhong wrote the original draft. All authors read and approved the final version of the manuscript, and ensure it is the case.

## CONFLICT OF INTEREST STATEMENT

The authors declare no conflict of interest.

## ETHICS APPROVAL AND CONSENT TO PARTICIPATE

The animal experiments were approved by the Ethics Committee of Nankai University (IACUC approval no. 2022‐SYDWLL‐000109), and the human studies were approved by the Medical Ethics Committee of Nankai University (approval no. NKUIRB2021015).

## CONSENT FOR PUBLICATION

Not applicable.

## Supporting information



Supporting Information

Supporting Information

Supporting Information

Supporting Information

Supporting Information

Supporting Information

Supporting Information

Supporting Information

Supporting Information

Supporting Information

Supporting Information

Supporting Information

## Data Availability

The experimental data presented in the study are included in the article/Supporting Information Materials, and further inquiries can be directed to the corresponding authors upon reasonable request.
