# Supplementary material for: Targeting the MDK/c‐Myc complex to overcome temozolomide resistance in glioma
Source: Clin Transl Med. 2025 Jun 4;15(6):e70359. doi: 10.1002/ctm2.70359 (PMC12137620; doi:10.1002/ctm2.70359)
Supplement: Supplementary file 1 — Supporting Information [file CTM2-15-e70359-s011.docx]

**Targeting the MDK/c-Myc Complex to Overcome Temozolomide Resistance in Glioma**

**Running title: Targeting the MDK/c-Myc complex to inhibit glioma**

Xiaonan Xi^1,2,5^, Xiaojing Ding^2^, Qianqian Wang^1,2^, Ning Liu^1,2^, Bangmao Wang^3^, Genbei Wang^5,*^, Weilong Zhong^3,*^ and Yaxin Lu^1,4,*^

^1^State Key Laboratory of Medicinal Chemical Biology, Nankai University, Tianjin 300350, China

^2^College of Pharmacy, Nankai University, Tianjin 300350, China

^3^Department of Gastroenterology and Hepatology, Tianjin Medical University General Hospital, Tianjin Institute of Digestive Diseases, Tianjin Key Laboratory of Digestive Diseases, Tianjin 300052, China.

^4^College of Chemistry, Nankai University, Tianjin 300350, China

^5^Pharmacology & Toxicology Research Center, National Key Laboratory of Chinese Medicine Modernization, Tasly Pharma Co., Ltd.，Tianjin 300410, China

*Correspondence to:

Yaxin Lu, State Key Laboratory of Medicinal Chemical Biology, College of Chemistry, Nankai University, Tianjin 300350, China. E‑mail: yaxinlu@nankai.edu.cn. Telephone: +86‑022‑23508470.

Weilong Zhong, Department of Gastroenterology and Hepatology, General Hospital, Tianjin Medical University, Tianjin 300052, China. E‑mail: zhongweilong@tmu.edu.cn. Telephone: +86‑022‑60361553.

Genbei Wang, Department of Pharmacology and Toxicology Research Center, National Key Laboratory of Chinese Medicine Modernization, Tasly Pharmaceutical Group Co., Ltd.，Tianjin 300410, China. E‑mail: wanggenbei@tasly.com. Telephone: +86‑022‑86342629.

**Funding**

The authors would like to thank the National Natural Science Foundation of China (Grant No. 82303509 and 82000511), Scientific and Technological Projects of Tianjin (21JCQNJC01120), Health Science and Technology Project of Tianjin (TJWJ2021QN006), Scientific Research Project of Tianjin Education Commission (2019KJ197), National key research and development program (2022YFC2504004), China Postdoctoral Science Foundation (2022M711711) for financially supporting this study.

**Author Contributions**

Yaxin Lu, Weilong Zhong, Genbei Wang and Xiaonan Xi designed this study. Xiaonan Xi and Xiaojing Ding performed the study. Qianqian Wang and Ning Liu was involved in the collection and analysis of proteomic data. Bangmao Wang participated in the analysis of clinical data. Xiaonan Xi and Weilong Zhong wrote the original draft. All authors read and approved the final version of the manuscript, and ensure it is the case.

**Corresponding authors**

Correspondence to Yaxin Lu, Weilong Zhong or Genbei Wang.

**Figure legends**

**Figure S1.** A-B. Validation of MDK expression in glial cell and glioma cell lines (PCR+WB). C. Detection of MDK expression at protein level and mRNA level after transfection with knockdown of MDK lentivirus. D. Detection of MDK expression at protein level and mRNA level after transfection with overexpressed MDK plasmid. E. The cells were inoculated into 24-well plates, and three holes were randomly selected according to the groups at intervals for cell count. The total number of cells in each hole was calculated, and the cell growth curve was drawn. Knockdown of MDK inhibited the proliferation and growth of cell lines with highly expressed MDK (SF126, U118MG, and U251). F. Growth detection of overexpressed-MDK cell lines (U87, SHG44, and BT-325). The overexpression of MDK promoted cell proliferation and growth. G. EDU assay was used to detect the proliferation ability of cell lines after MDK knockdown (SF126, U118MG, and U251). H. The proliferation capacity of MDK overexpression cell lines was detected by the EDU method (U87, SHG44, and BT-325). I. Transwell assay was used to detect the invasion and migration ability of the cell lines (SF126, U118MG, and U251) after MDK knockdown. J. The invasion and migration ability of MDK-overexpression cell lines (U87, SHG44, and BT-325) were detected by Transwell assay. K. The proliferation ability of MDK knockdown cells (SF126, U118MG, and U251) was detected by clone formation assay. L. The number of clones formed in the U87, SHG44, and BT-325 cell lines after the overexpression of MDK was measured by clonal formation assay. M. Cell proliferation assay of HEB cells with induced MDK expression. N. Colony formation assay of HEB cells with induced MDK expression. O. Cell invasion assay of HEB cells with induced MDK expression. Error bars represent mean ± SD, *P < 0.05, **P < 0.01.

**Figure S2.** A. Cell lysates from overexpressing MDK U87 cells were immunoprecipitated and immunoblotted with antibodies against the indicated proteins. B. The cell lysate of U87 cells that did not overexpress MDK were immunoprecipitated and immunoblotted with antibodies against the indicated proteins. C. Co-localization of c-Myc and MDK by immunofluorescence staining in U118MG cells.

**Figure S3.** U87, SHG44, BT-325, SF126, U118MG and U251 cells were transfected with MDK-overexpression plasmids and then treated with IWR-1 (10 μM) or adavivint (100 nM) for 24 h. A–C. Cell viability assay of U87, SHG44, BT-325, SF126, U118MG and U251 cells. D–F. EdU assay of cell viability in the cell lines. G–I. Transwell assay of cell invasion ability in the cell lines. J-L. The rescue experiments were performed by overexpressing c-Myc in MDK-knockdown cells. Edu and Transwell methods were used to detect cell proliferation and invasion in SF126 (J), U118MG (K), U251 (L). Error bars represent mean ± SD, *P < 0.05, **P < 0.01.

**Figure S4.** A. After 72 h of TMZ treatment, cell proliferation capacity was measured by EdU method. B. After incubation with TMZ for 48 h, the invasive ability of the cells was detected by the Transwell method. C. Colony formation assay of proliferation ability in SF126 cells. In the TMZ group, after 48 h of TMZ treatment, the medium was changed until the monoclone was formed. Error bars represent mean ± SD, *P < 0.05, **P < 0.01.

**Figure S5.** The SF126 cells were incubated with 5 μM ACT001 or equivalent PBS for 72 h and treated with 10 μM MG132 for 4 h. After the cells were collected, they were immunoprecipitated using c-Myc antibodies and immunoblotted with K48 or K63 linked ubiquitination antibodies.

**Figure S6.** A. After treatment with ACT001 at different concentrations for 48 h, the cell proliferation capacity was detected by the EdU method. B. Transwell assay results of cell invasion ability after incubation with different concentration of ACT001 for 48 h. C. The effects of temozolomide and ACT001 on the proliferation of glioma cell lines were investigated by a clonal formation assay. The drug was incubated for 48 h and then replaced with a new medium until it grew into a monoclone. D. Western blot analysis of c-Myc expression, Wnt/β-catenin signaling pathway and EMT marker expression in the SF126 and U118MG cells treated for 48 h under different conditions.

**Figure S1**

**
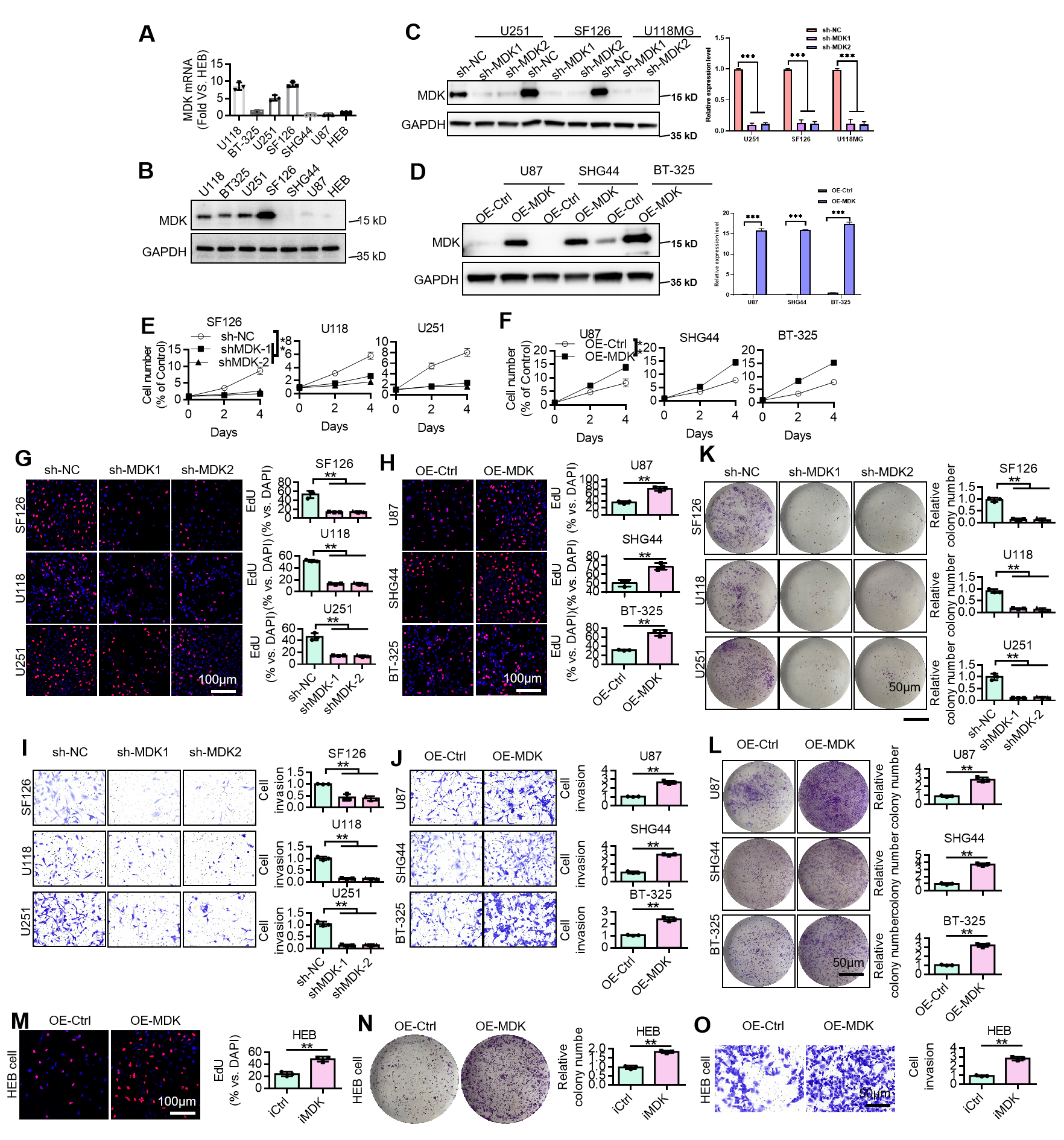
**

**Figure S2**

**
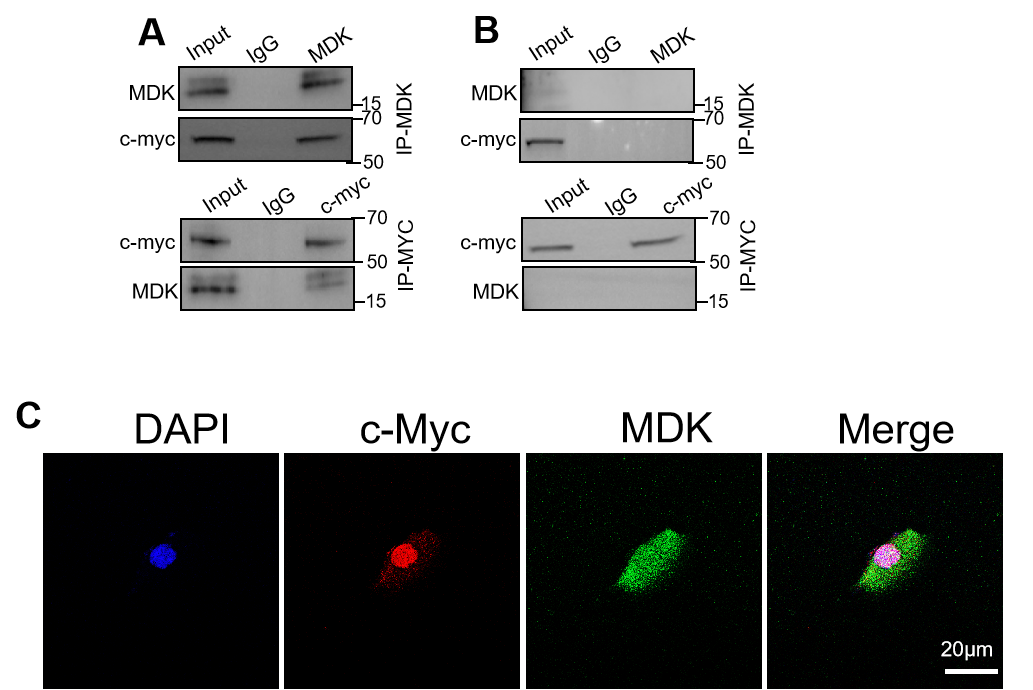
**

**Figure S3**

**
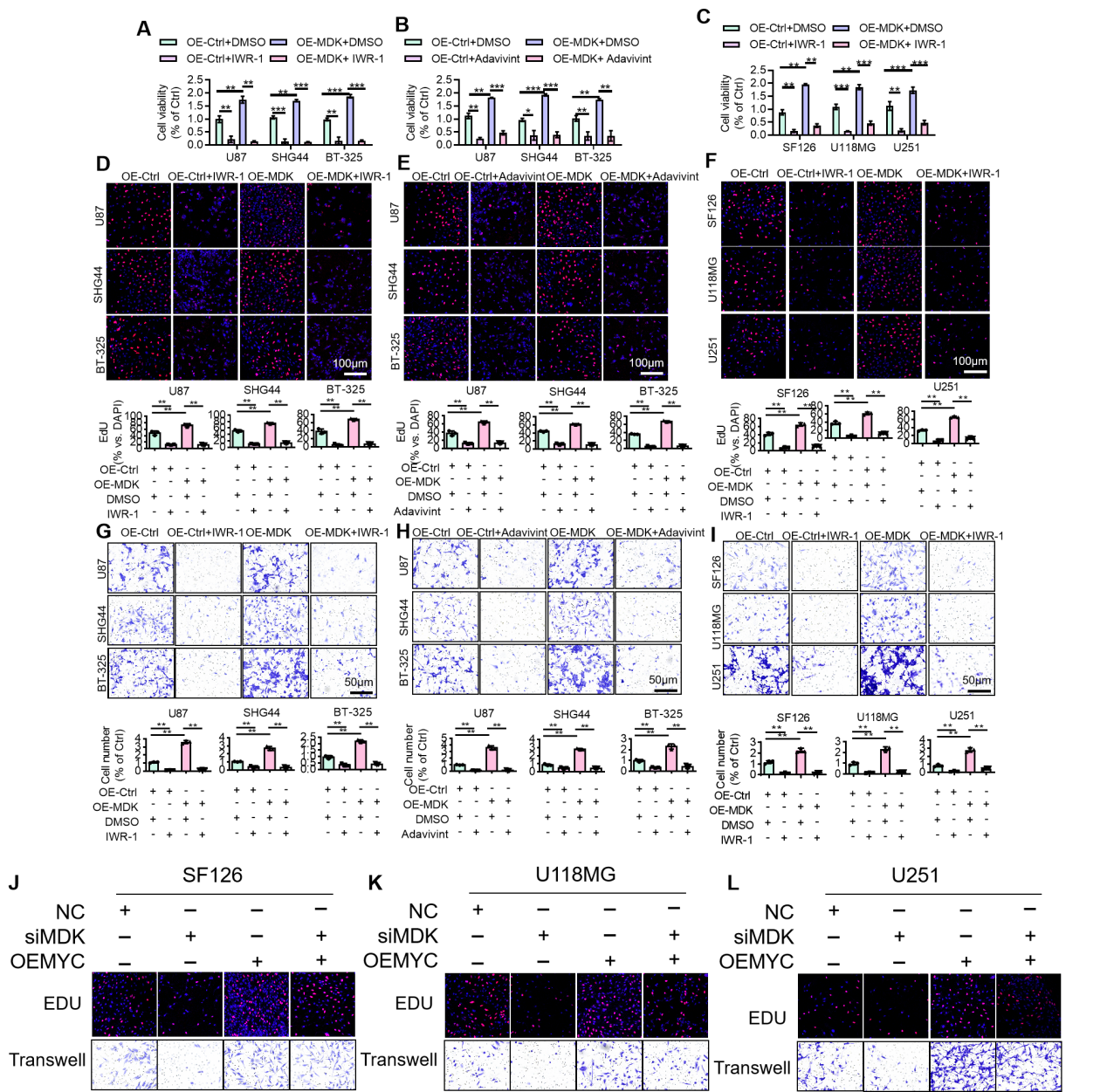
**

**Figure S4**

**
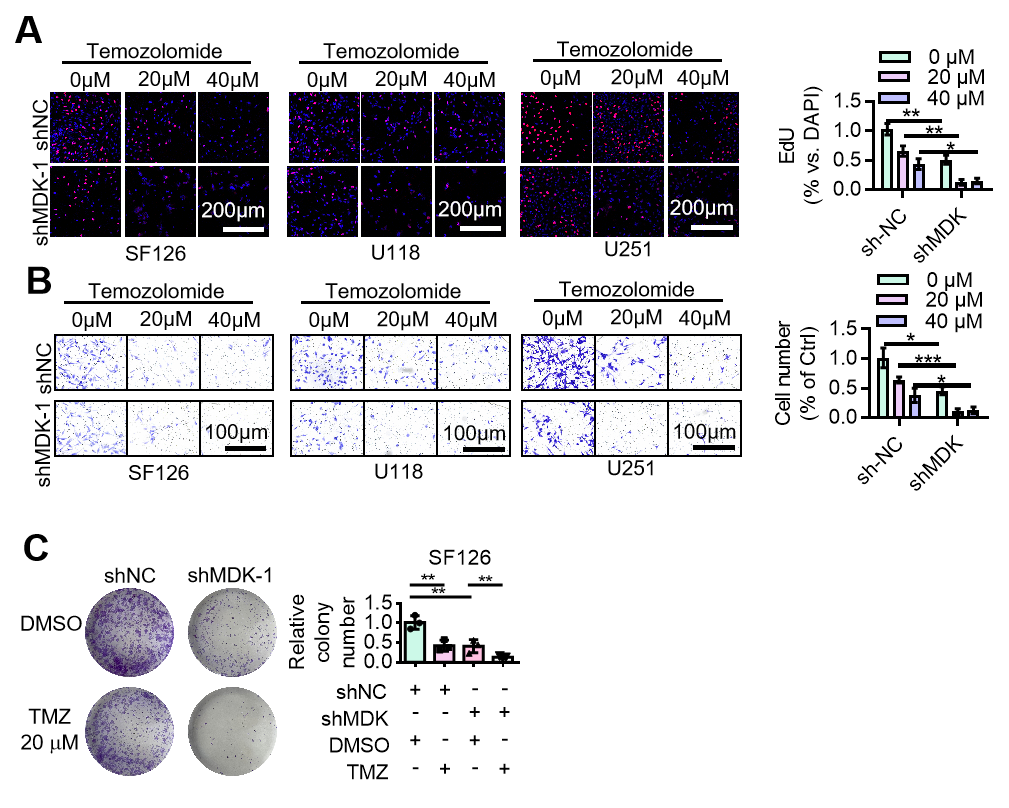
Figure S5**

**
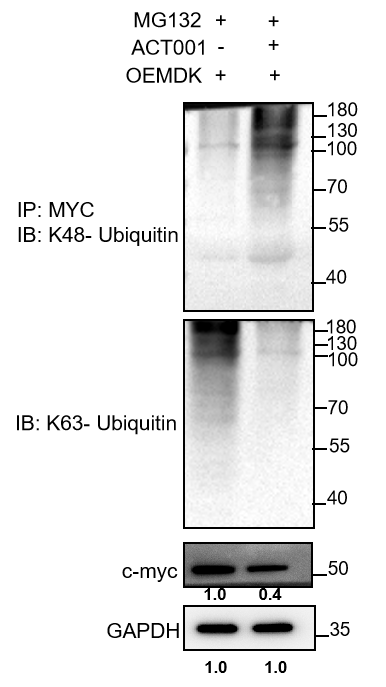
**

**Figure S6**

**
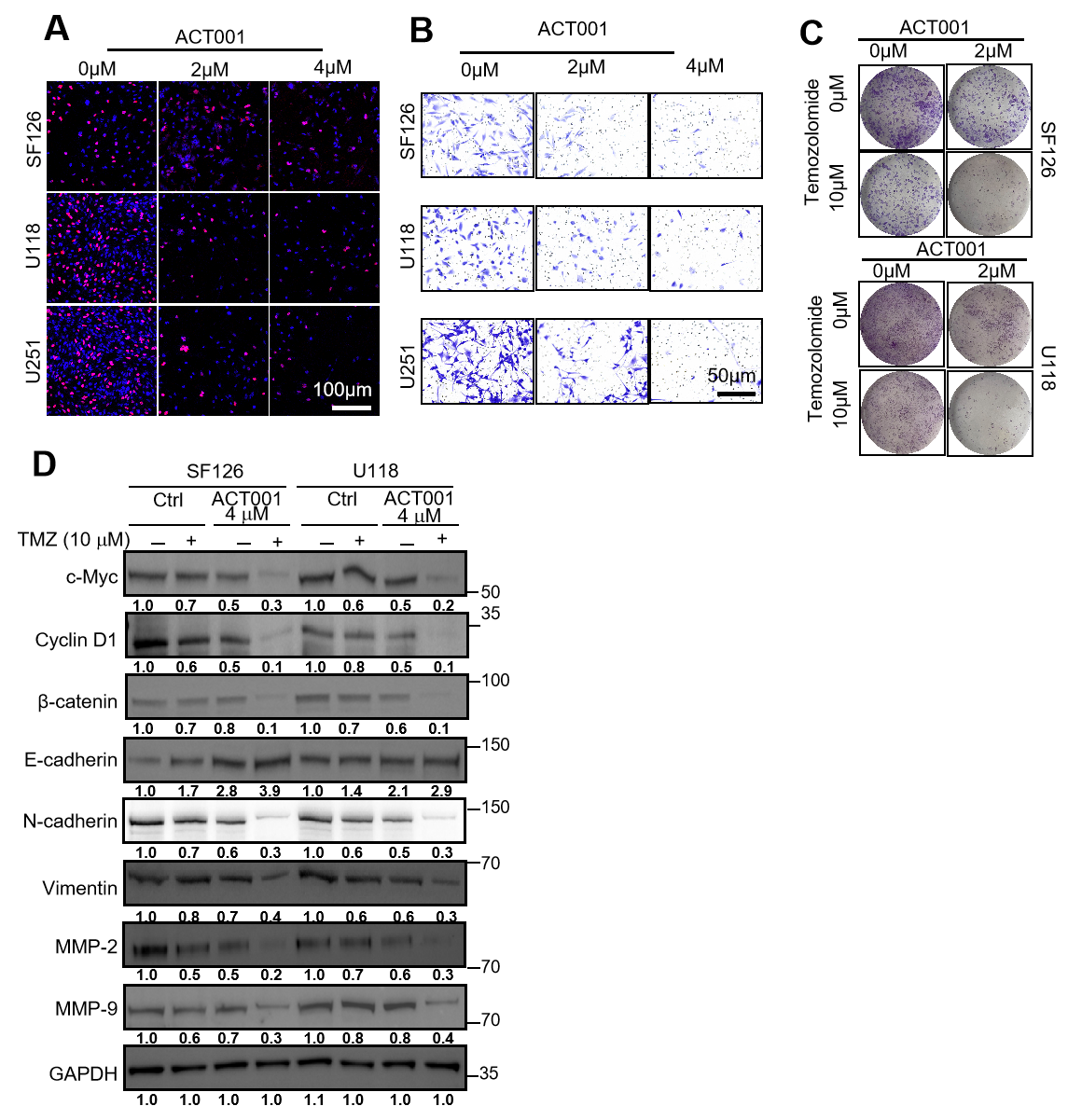
**
